# Organic Phase Change Materials for Thermal Energy Storage: Influence of Molecular Structure on Properties

**DOI:** 10.3390/molecules26216635

**Published:** 2021-11-02

**Authors:** Samer Kahwaji, Mary Anne White

**Affiliations:** 1 Department of Chemistry, Dalhousie University, Halifax, NS B3H 4R2, Canada; sam@dal.ca; 2 Department of Chemistry and Clean Technologies Research Institute, Dalhousie University, Halifax, NS B3H 4R2, Canada

**Keywords:** phase change materials, melting, thermodynamics, alkyl chains, thermal energy storage

## Abstract

Materials that change phase (e.g., via melting) can store thermal energy with energy densities comparable to batteries. Phase change materials will play an increasing role in reduction of greenhouse gas emissions, by scavenging thermal energy for later use. Therefore, it is useful to have summaries of phase change properties over a wide range of materials. In the present work, we review the relationship between molecular structure and trends in relevant phase change properties (melting temperature, and gravimetric enthalpy of fusion) for about 200 organic compounds from several chemical families, namely alkanes (paraffins), fatty acids, fatty alcohols, esters, diamines, dinitriles, diols, dioic acids, and diamides. We also review availability and cost, chemical compatibility, and thermal and chemical stabilities, to provide practical information for PCM selection. Compounds with even chain alkyl lengths generally give higher melting temperatures, store more thermal energy per unit mass due to more efficient packing, and are of lower cost than the comparable compounds with odd alkyl chains.

## 1. Introduction

Almost half of the world’s energy demand is used for heating [1], yet more than 60% of the global energy demand ultimately becomes dissipated as waste heat [2]. This mismatch situation significantly contributes to global climate change, but also offers an opportunity for considerable improvement if waste heat can be stored for later use.

Thermal energy storage can take place via the specific heat capacity of a material, such as brick or water, via so-called sensible storage. Typical values of sensible heat storage are 0.92 J K^−1^ g^−1^ (brick) and 4.2 J K^−1^ g^−1^ (water) [3], which correspond to gravimetric values of 1.6 J K^−1^ cm^−3^ (brick) and 4.2 J K^−1^ cm^−3^ (water), which are of more practical importance. Therefore, 1 g of brick can store 0.92 J over a 1 K temperature rise, whereas 1 g of water can store 4.2 J over a 1 K temperature rise, via sensible storage.

However, if the energy needed to be stored at ca. 0 °C, advantage could be taken of the enthalpy of fusion (latent heat) of H_2_O, which is 334 J g^−1^. Heating 1 g of H_2_O from −0.5 °C to 0.5 °C would make use of both the sensible heat storage (4.2 J; already large due to the high specific heat of water) and the significantly higher value of the latent heat (334 J). Relative to its sensible heat storage properties alone, the required mass (or volume) of water could be reduced by almost two orders of magnitude by making use of water’s abilities as a phase change material (PCM).

At its melting point, the latent heat of H_2_O (334 J g^−1^) provides energy storage of 93 Wh kg^−1^, which places H_2_O in the gravimetric energy density range of nickel metal hydride batteries, and only 30% lower than some lithium-ion batteries [4]. Phase change materials with more modest latent heats of 100 J g^−1^ are still in the energy density range of lead acid batteries [4]. However, we make this comparison only for illustration of the potential of phase change materials, mindful that batteries and phase change materials store different forms of energy and have different exergy densities and efficiencies.

Of course, the phase change material only has such a significant thermal energy storage capacity in the range of the transition temperature, and therefore different PCMs with different melting points would need to be used for different applications. Other requirements for PCMs include high enthalpy of fusion, small volume change, excellent reproducibility over many melt-freeze cycles, absence of hysteresis, low cost, safe for use, and high thermal conductivity. It is difficult to achieve all of these properties in a given PCM, so compromise might be required. Nevertheless, a recent Bloomberg report assesses the phase change materials market at more than USD 4 billion by 2024 [5].

For low to moderate temperatures, select organic molecular solids can have favorable enthalpies of fusion, can melt and freeze reproducibly, and can be safe and cost effective. Examples include alkanes (paraffins), alcohols, fatty acids, and esters. For higher temperatures, inorganic materials including salt hydrates are generally preferred. For reviews of phase change materials and their applications, see elsewhere [6,7,8].

In a recent study [9], we investigated the thermodynamics of fusion (melting) for nearly 7000 organic compounds, with an emphasis on those with unusually high changes in entropy on fusion (Δ_fus_*S* > 85 J K^−1^ mol^−1^). The large values of Δ_fus_*S* correlated with favorably high enthalpy changes (Δ_fus_*H* = *T*_fus_ Δ_fus_*S*). Many of the compounds with high Δ_fus_*S* were nonrigid molecules, and, on melting, the flexibility of the molecules gave rise to degrees of freedom in addition to the usual onset of translational motion, and therefore provided a higher Δ_fus_*S* than for rigid molecules. Many molecules with potential as PCMs also had extensive hydrogen bonding: their melting point is generally higher than similar molecules without hydrogen bonding, leading to high values of Δ_fus_*H,* even if Δ_fus_*S* is in the normal range. Therefore, molecular solids in which the molecules are flexible and/or H-bonded, are strong prospects for phase change materials based on their high values of Δ_fus_*H.*

However, there is scope to learn more from studies of melting of long-chain unbranched organic molecules, given their role as phase change materials. In the present review, we focus on trends in melting points and enthalpies of fusion (expressed in the more practical terms of J g^−1^, not the more theoretically important J mol^−1^) for several families of organic molecules with potential applications as PCMs.

## 2. Trends Based on Length of Alkyl Chain

Many of the organic compounds with flexible molecules that could have high values of Δ_fus_*H* have molecular motifs with long alkyl chains. In the present review, we concentrate on linear (unbranched) chains in the following materials: alkanes (paraffins; general formula C*_n_*H_2*n*+2_); fatty acids of the general formula CH_3_(CH_2_)*_n_*_−2_COOH; fatty alcohols of the general formula CH_3_(CH_2_)*_n_*_−2_CH_2_OH; esters of the general formula CH_3_(CH_2_)*_m_*_−1_-O-C(O)-(CH_2_)*_n_*_-*m*−2_CH_3_; diamines of the general formula H_2_N-(CH_2_)_n_-NH_2_; dinitriles of the general formula NC-(CH_2_)*_n_*_−2_-CN; diols of the general formula HO-(CH_2_)*_n_*-OH; dioic acids of the general formula HOOC(CH_2_)*_n_*_−2_COOH; and diamides of the general formula H_2_N-C(O)-(CH_2_)*_n−_*_2_-C(O)-NH_2_. (The last five are α,ω-disubstituted alkanes.) These materials are our focus based on practical considerations, including their high Δ_fus_*H* and relatively low cost for large scale use as phase change materials. Sugar alcohols have high enthalpy changes but are subject to significant supercooling [10], which makes it difficult to recover thermal energy stored through their melting transition. Other organic compounds, such as aliphatic fatty diamides [11], fatty amides [12] and aromatic esters [13], also hold promise as phase change materials. 

We begin with consideration of the melting points as a function of compound type and number of carbons in the structure.

### 2.1. General Trends in Melting Points 

**Figure 1:**
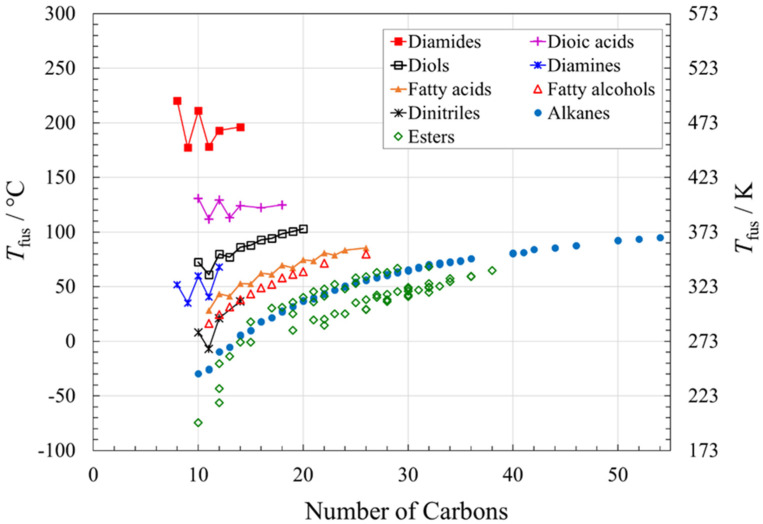
Melting points of nine families of long-chain, unbranched organic molecular solids, as a function of chemical family and total number of carbons in the compound. Lines are drawn between data points for some families, to emphasize odd–even effects, as described in the text. See Supplementary Information for data and data sources.

Figure 1 shows the melting points (*T*_fus_) for a large number of long-chain organic compounds, by compound type and by number of carbons in the alkyl chain(s), with data from extensive compilations [14,15,16,17,18,19,20,21,22,23,24,25,26,27,28,29,30,31,32,33,34,35,36,37,38,39,40,41,42,43,44,45,46,47,48,49,50,51,52,53,54,55,56,57,58,59,60,61,62,63,64,65,66,67,68,69,70,71,72,73,74,75,76,77,78,79,80,81,82,83,84]. For detailed sources by compound and all data used in the present analyses, see Supplementary Information.

The highest melting points at a given number of carbons in the backbone of the compounds considered occur for the diamides, due to their very strong hydrogen bonding interactions. H-bonding per molecule decreases on moving to dioic acids, then diols and diamines, then fatty acids and fatty alcohols and dinitriles, then alkanes and esters, and the melting point for a given number of carbons decreases accordingly. Alkanes and esters have the lowest melting points for a given number of carbons, reflecting their lack of H-bonds (alkanes) or weak (esters) H-bonding.

### 2.2. Melting Points as a Function of Carbons in Backbone

We again refer to Figure 1, which shows melting point trends as a function of the number of carbons in the backbone. For all nine compound types considered, the general trend within a family is to higher melting point at higher molecular mass, given the greater thermal energy required to melt larger molecules. However, when the situation for a given family is looked at in more detail, there are some exceptions to the trend.

**Figure 2:**
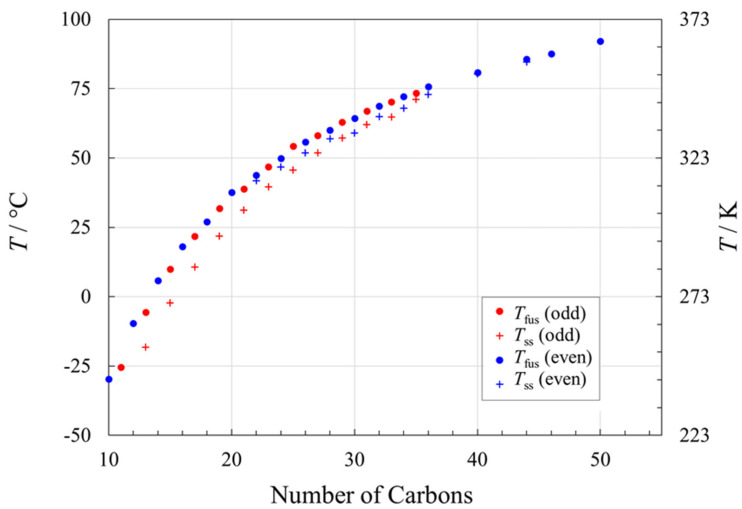
Phase transitions in *n*-alkanes (paraffins) as a function of chain length, *n*. Circles (● for even *n*; ● for odd *n*) represent melting temperatures, and crosses (+ for even *n*; + for odd *n*) represent the temperatures of the main solid–solid transition, if present. See Supplementary Information for data and data sources.

For the alkanes (paraffins), close examination of the melting points as a function of chain length (Figure 2) reveals two nearly parallel trends for *n* < 20, one for the even numbers of carbons, and one for the odd chain lengths [19,26,27]. These separate trends arise from their two distinct packing arrangements [85,86], with even-carbon chains packing more efficiently and therefore having higher melting points [16]. For 20 < *n* < 44, the even-carbon alkane series also has one or more solid–solid transitions before the melting point [26,87,88]. Similarly, solid–solid transition(s) occur in the odd-carbon alkanes for 11 > *n* > 41 [26,87,88]. The solid–solid transitions pre-empt some of the enthalpy change that otherwise would be used for Δ_fus_*H*, as discussed below.

Long-chain alcohols can exhibit at least three types of crystalline forms [87], and again have different packing of the odd and even alkyl chains. Although the solid–solid transition temperatures of long-chain alcohols show marked odd–even effects [87], the melting points (Figure 1) show a nearly smooth curve. 

Fatty acids with even chain lengths exist as one of three polymorphs in the solid state, differentiated by the tilt angle of the carboxyl headgroup with respect to the alkyl tail. The melting points of the fatty acids again exhibit strong odd–even effects (Figure 1), due to the different packings of even and odd alkyl chains [85,86]. 

**Figure 3:**
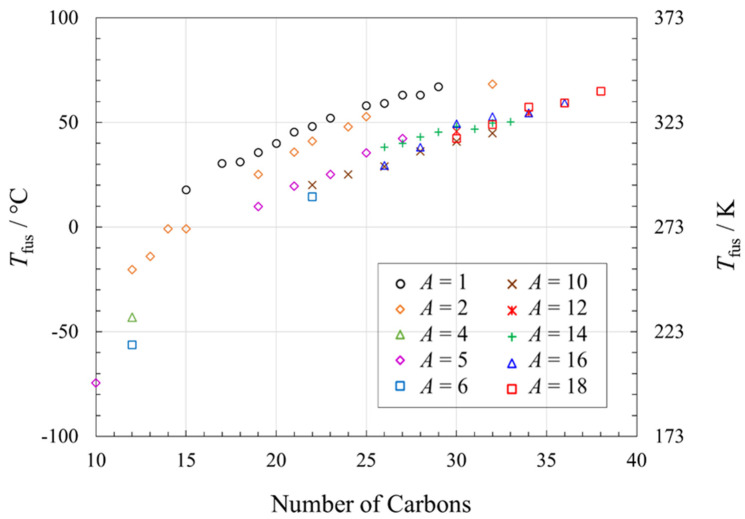
Melting points of esters of general formula CH_3_(CH_2_)*_m_*_−1_-O-C(O)-(CH_2_)*_n_*_-*m*−2_CH_3_ where *A* is the length of the alkyl chain (*A* = *m*), as a function of the total number of carbons. See Supplementary Information for data and data sources.

Melting point trends in esters as a function of total number of carbons are difficult to discern from Figure 1, due to the large number of isomers. However, our review of melting data [9,22,39,40,41,42,43,44,45,46,47,48,49,50,51,52,53,54,55,56,57] by type of ester, is more revealing (Figure 3), where *A* is the alkyl chain length (*m* in the general formula CH_3_(CH_2_)*_m_*_−1_-OC(O)-(CH_2_)*_n_*_-*m*−2_CH_3_). Within a given family, such as methyl esters (*A* = 1), the melting point increases with the total number of carbons in the compound. As we showed in another more limited investigation, with a given number of carbons in the ester, the shorter the alkyl chain, the higher the melting point [9], as is now shown here to be quite general. For a given overall number of carbons, methyl and ethyl esters have higher and similar melting points, and esters with longer alkyl chains have lower (and similar) melting points. Shorter alkyl chains (i.e., structures with the ester group at the end of the chain such as methyl esters) have higher melting points than for their isomers with the ester functional group in the middle of the chain, due to the rotational flexibility of the O-C bond along the chain, which can disrupt the lattice and lower the melting point. This rotational flexibility has more influence when the ester group is not at the alkyl chain end of the molecule, and therefore methyl esters have the highest melting points [13].

Figure 1 also reveals odd–even effects in diamines, dinitriles, diols, dioic acids, and diamides, again with the odd chains at lower melting points, presumably due to their less efficient packing and therefore weaker intermolecular interactions.

## 3. Enthalpies of Fusion

### 3.1. Overview

**Figure 4:**
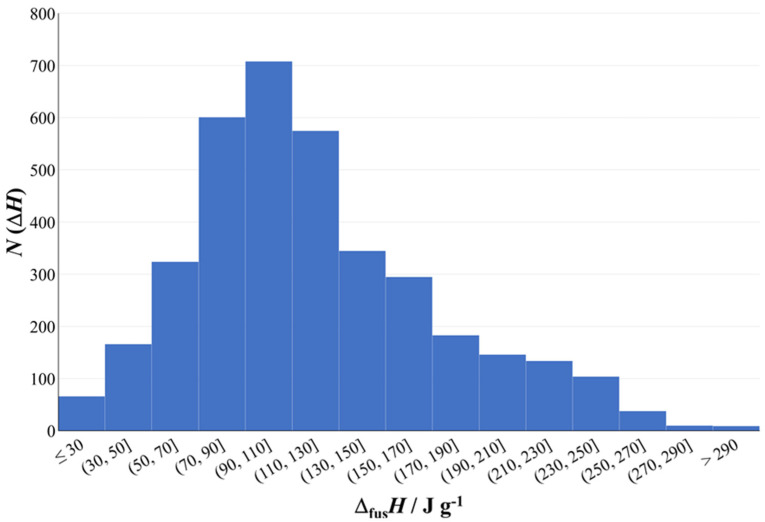
Number of molecular organic compounds with a given range of gravimetric enthalpy of fusion, Δ_fus_*H* expressed in J g^−1^, based on published summaries of literature data [14,15].

Making use of the published extensive summaries of literature data [14,15], we have assessed the values of Δ_fus_*H*, expressed in J g^−1^, for nearly 7000 organic compounds. The overall results are shown in Figure 4. 

We chose to use the gravimetric values (i.e., per unit mass) rather than the molar values, as the former is more representative of the requirements for practical applications as phase change materials. Note that the densities of most of these organic compounds are very similar (ca. 0.9 g cm^−3^; an exception is sugar alcohols with densities of ~1.5 g cm^−3^, contributing to their exceptionally high Δ_fus_*H*), so trends in gravimetric values of Δ_fus_*H* also pertain to volumetric values for the molecules considered here.

Most of these ~7000 organic compounds, the families of which extend far beyond the nine considered in detail here, have Δ_fus_*H* values of ~100 J g^−1^. This range would be suitable for use as phase change materials and provides an energy density of ca. 30 Wh kg^−1^ which is comparable to some batteries, but higher values of Δ_fus_*H* are better. Note again that Δ_fus_*H* for water is 334 J g^−1^, which is exceptionally high due to H_2_O’s relatively high melting point (273 K; despite the lower molar mass, the H_2_O melting point is higher than H_2_S [187 K] and H_2_Se [208 K] due to strong hydrogen bonding in H_2_O). Therefore, H_2_O offers a unique opportunity for use as a PCM near 273 K. Nevertheless, based on Figure 4, there are a number of organic compounds with Δ_fus_*H* values of >200 J g^−1^. Detailed consideration of the compounds in this category again reveals the importance of flexibility of the molecules and the importance of hydrogen bonding. Further detailed analysis here is confined to the nine families of compounds described above, which exemplify these features.

### 3.2. Alkanes 

**Figure 5:**
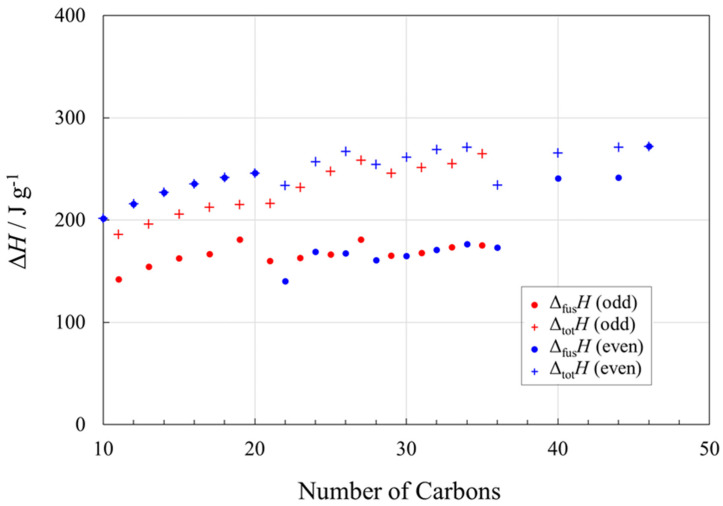
Enthalpy changes (Δ*H* in J g^−1^) associated with phase transitions in *n*-alkanes (paraffins), as a function of chain length, *n*. Circles (● for even *n*; ● for odd *n*) represent the enthalpies of fusion, Δ_fus_*H*, and crosses (+ for even *n*; + for odd *n*) represent the total enthalpy changes of fusion plus that from solid–solid transition(s), if present, Δ_tot_*H*. For even *n* ≤ 20, Δ_fus_*H* = Δ_tot_*H*. See Supplementary Information for data and data sources.

Alkanes (paraffins) are among the most prominent PCMs used commercially, in applications including residential solar thermal systems and passive thermal management systems. From Figure 5, it is apparent that the values of Δ_fus_*H* are favorable for thermal energy storage (>100 J g^−1^) and that the odd–even chain length effect observed in the alkane melting points (Figure 2) is even more prominent in Δ_fus_*H*.

We consider the values of Δ*H* for alkanes in three segments, according to the range of the chain length, *n*.

For *n*-alkanes where *n* is even and ≤20, there are no solid–solid transitions (or they are not discernible from the available data) and Δ_fus_*H* = Δ_tot_*H*. On a gravimetric basis, these alkanes have much higher values of Δ_fus_*H* than for odd values of *n*, largely because the latter have one or more solid–solid phase transitions below the melting point. However, even if the enthalpy change associated with the solid–solid transition(s), which is typically ~10 K below the melting point, is included, the total enthalpy change for odd values of *n* is less than for even values. This situation likely arises due to more efficient packing of the even chains, and the packing is disrupted more on melting (higher Δ_fus_*S* so higher Δ_fus_*H*) than for the odd chains.

For even values of *n* from 22 to 36, Δ_fus_*H* generally follows the lower Δ_fus_*H* trend of the odd values of *n*, and a major contributing factor is that there is at least one solid–solid transition for the even *n* values at these chain lengths. There is a 40% drop in Δ_fus_*H* from *n* = 20 with no solid–solid transition (Δ_fus_*H* = Δ_tot_*H* = 246 J g^−1^) to *n* = 22 (Δ_fus_*H* = 140 J g^−1^). However, docosane (C_22_H_46_) has a solid–solid transition that is only about 2 K lower in temperature than its melting point, and the total enthalpy change (Δ_s-s_*H* + Δ_fus_*H*) is 234 J g^−1^ [27]. The solid–solid transition in triacontane (C_30_H_62_) is about 5 K lower than its melting point and its corresponding enthalpy change is ca. 60% of the value of Δ_fus_*H*. 

For higher values of *n*, e.g., *n* = 46, there are no solid–solid transitions [19].

Note that if the solid–solid transition(s) in a long-chain alkane used as a PCM is/are outside the temperature region of energy storage/retrieval, then only Δ_fus_*H* is available. In such a case of a narrow operational temperature, alkanes with even *n* and *n* < 20 or *n* > 44 would be preferable. For a wider operational temperature range, other *n* values would still be useful, although alkanes with longer chains and odd values of *n* are more expensive as we discuss below. In practical terms, latent heat thermal energy storage modules require a temperature difference of 5 to 10 K (=5 to 10 °C) between the PCM and the heat transfer fluid to work efficiently, and if the solid–solid transitions are within this range then they should be included in the energy balance of the system.

### 3.3. Fatty Alcohols

**Figure 6:**
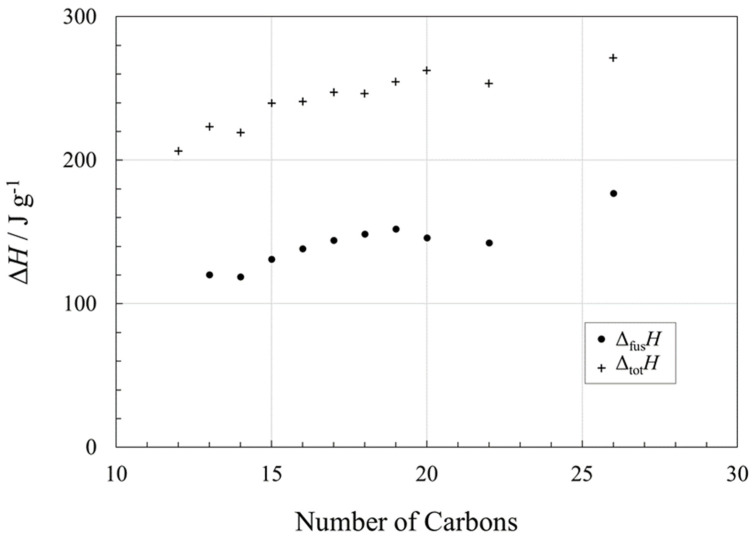
Enthalpy changes (Δ*H* in J g^−1^) associated with phase transitions in linear 1-alcohols, as a function of chain length, *n*. Circles (●) represent the enthalpies of fusion, Δ_fus_*H*, and crosses (**+**) represent the total enthalpy changes of fusion plus that from solid–solid transition(s), if present, Δ_tot_*H*. See Supplementary Information for data and data sources.

The enthalpies of fusion of long chain saturated fatty alcohols as a function of chain length are shown in Figure 6. These show only very subtle odd–even effects, certainly much smaller than in the alkanes.

Solid–solid transitions in the long-chain alcohols take place prior to melting [70] and they can have considerable associated enthalpy changes [15]. However, the difference between the temperature of the solid–solid transition(s) and the melting point are generally very small, ~3 K for 1-decanol, and ~1 K for 1-dodecanol, 1-tetradecanol, 1-hexadecanol, and 1-octadecanol [89]. As for the alkanes, the full enthalpy change (Δ_s-s_*H* + Δ_fus_*H*) can be accessed for thermal energy storage if the PCM operational temperature range is sufficiently broad.

For a given melting point (Figure 1), Δ_fus_*H* in J g^−1^ of a fatty alcohol is similar to that for the corresponding alkane, although the alcohol would have a shorter chain length. Therefore, use of an alcohol could provide an economic benefit. 

### 3.4. Fatty Acids

Linear saturated fatty acids with even numbers of carbon atoms in the chain, form one of three polymorphs (A, B, C) in the solid phase, with subtle differences in tilt angle and the head group relative to the tail [90]. The C phase grows from the melt and there can be subtle solid–solid transitions, such as the one at ~200 K in dodecanoic acid [91]. 

**Figure 7:**
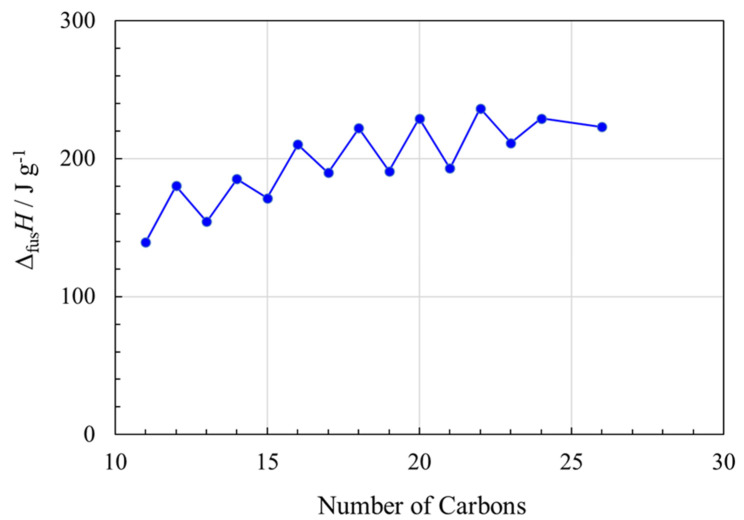
Enthalpy changes (Δ*H* in J g^−1^) associated with fusion (melting) of linear saturated fatty acids, as a function of number of carbons in the chain, *n*. See Supplementary Information for data and data sources.

The enthalpies of fusion of long chain fatty acids as a function of chain length are shown in Figure 7. There are noticeable odd–even effects, again with higher values of Δ_fus_*H* for even numbers of carbons, again likely due to their more efficient packing. Since any solid–solid transitions are subtle and at very much lower temperatures, we do not include them in this analysis.

For a given chain length, Δ_fus_*H* in J g^−1^ of a fatty acid with *n* < 20 is a little less than for the corresponding alkane, but for *n* > 20, Δ_fus_*H* of the fatty acids is higher than Δ_fus_*H* for the alkanes, and about the same as Δ_tot_*H* for the alkanes, i.e., including their solid–solid transition(s). The order of the chain length that melts at a given temperature, from shortest to longest, is fatty acid < alcohol < alkane (Figure 1). 

### 3.5. Esters

**Figure 8:**
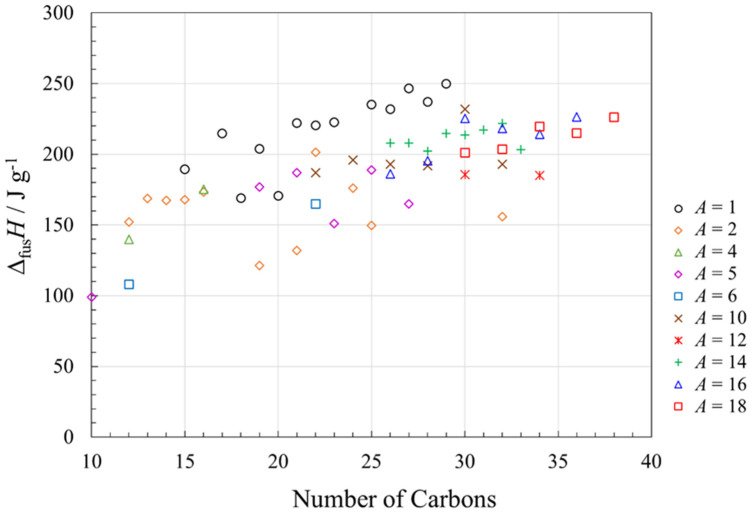
Gravimetric enthalpy changes (Δ*H* in J g^−1^) associated with fusion (melting) of linear saturated esters of general formula CH_3_(CH_2_)*_m_*_−1_-O-C(O)-(CH_2_)*_n_*_-*m*−2_CH_3_ where *A* is the length of the alkyl chain (*A* = *m*), as a function of the total number of carbons in the molecular structure. See Supplementary Information for data and data sources.

In our previous investigation of enthalpy changes due to melting in long-chain saturated esters, trends in Δ_fus_*H* were not very clear [9]. Several matters advance the analysis in the present review: (a) the publication of new melting studies of esters [17,39,45]; (b) the presentation of the present results in terms of gravimetric (J g^−1^) enthalpy of fusion, not molar (J mol^−1^) values; and (c) our analysis of the literature data for accuracy (the data from Acree and Chickos [14,15] are thoroughly tabulated but not assessed for accuracy). The present analysis of gravimetric enthalpies of fusion for long-chain saturated esters is shown in Figure 8.

The gravimetric enthalpies of fusion show a general trend to higher values as the number of carbons in the molecular structure increases. Within a given alkyl chain length (*A*), there are discernible odd–even effects (especially apparent for methyl esters, *A* = 1), with the odd values giving lower values of Δ_fus_*H*. This trend also was observed for alkanes (Figure 5) and fatty acids (Figure 7) and again can be associated with different packing regimes for odd and even chain lengths [17], although the ester melting points (Figure 3) do not show very large odd–even effects. For a given number of carbons in the ester backbone, the general trend is to higher values of Δ_fus_*H* with shorter alkyl chains (smaller *A* values), but these trends are not always followed (e.g., for *n* = 22, the order of decreasing Δ_fus_*H* is *A* = 1, 2, 10, 6). With the wide Δ_fus_*H* range of isomeric esters, i.e., esters with the same total number of carbons, it is difficult to make comparisons of enthalpies of fusion with alkanes, alcohols and fatty acids at the same overall chain length, or at a given melting point. However, the values of Δ_fus_*H* for the vast majority of the esters examined are >150 J g^−1^, which is very favorable for their use as phase change materials.

### 3.6. α,ω-Disubstituted Alkanes

**Figure 9:**
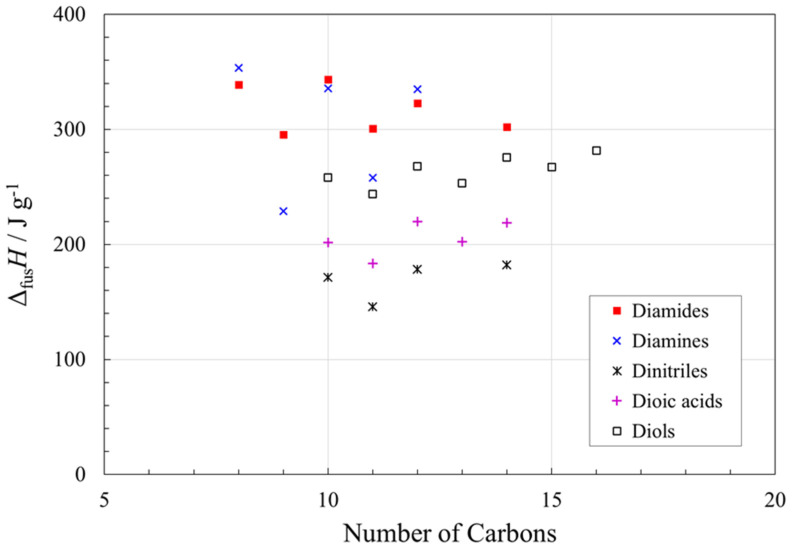
Gravimetric values of Δ_fus_*H* for diamines, dinitriles, diols, dioic acids and diamides (all are α,ω-disubstituted alkanes), as a function of number of carbons in the molecule. See Supplementary Information for data and data sources.

The five families of α,ω-disubstituted alkane compounds, namely diamines, dinitriles, diols, dioic acids and diamides, all show quite high gravimetric values of Δ_fus_*H* (Figure 9), and also show odd–even effects, again with the higher values of Δ_fus_*H* for even carbon numbers. (Note that nonane-1,9-diamine has a small solid–solid transition ca. 7 K below its melting point, with Δ_s-s_*H* about 20% of Δ_fus_*H* [82]. Also, some of the diamides show solid–solid transitions, but much below their melting points [18] and are not included in Δ*H* shown in Figure 9).

Most remarkable are the high gravimetric Δ_fus_*H* values of the diamines and diamides, especially with even numbers of carbons, even exceeding the value for water (334 J g^−1^) in the cases of octane−1,8-diamine at 353 J g^−1^ [82] and suberamide (*n* = 8) at 339 J g^−1^ [18]. The relatively strong hydrogen bonding at both ends of these molecules and flexibility of the alkyl chains contribute to these exceptionally high values of Δ_fus_*H.* A thorough study of diamides showed that only some short-chain compounds decompose on melting [18]. Within the α,ω-disubstituted alkane compounds considered here, dinitriles have the lowest values of gravimetric Δ_fus_*H*, reflecting their low contribution from hydrogen bonding.

## 4. Selection of a Phase Change Material

### 4.1. Melting Temperature

The most important attribute of a PCM is that its transition temperature (melting point/freezing point) should match the temperature range of the application. 

Among the molecular, organic phase change materials discussed here, the melting points vary from −70 °C to ca. 200 °C, which allows many opportunities for PCMs that meet phase transition temperature requirements. At any given phase transition temperature, there are often several choices among these organic PCMs, which we now consider further.

### 4.2. Energy Storage and Costs

A phase transition temperature of ca. 50 °C, for example, could be achieved with a diamine (*n* = 8), fatty acid (*n* =14), alcohol (*n* =16), alkane (*n* = 22), or various esters. The values of enthalpy of fusion indicate that to store a given quantity of thermal energy, the required mass would in this case be lowest for the diamine, and about twice as high for the alkane, fatty acid or alcohol. However, based on price, where all prices have been assessed via the lowest metric ton prices as listed on the website of a bulk materials company that ships internationally [92], for storage of a given quantity of thermal energy, the fatty acid (at USD 500 per metric ton) would be the least expensive, the alcohol would be about four times that cost, and the alkane and diamine would be nearly 30 times that cost.

As a second example, we consider a phase transition temperature of ca. 70 °C which could be met with a diol (*n* = 10), a diamine (*n* = 12), a fatty acid (*n* =18), a fatty alcohol (*n* = 22), or an alkane (*n* = 34). The values of enthalpy of fusion indicate that in this case the required mass would again be lowest for the diamine, and 30–50% higher for the alcohol, diol and fatty acid, and about twice as high for the alkane. More telling is the absence of pricing at metric ton quantities for any of the above, except for the fatty acid at USD 600 per metric ton [92], indicating that the diol, diamine, fatty alcohol and alkane are not commodity items.

Another important point concerning pricing is whether the organic phase change material has an even or odd number of carbons. As we have discussed already, even-carbon compounds tend to have higher melting points, which means the even-carbon compounds have melting points corresponding to longer chains for odd numbers of carbons. This is a valuable asset for even carbon compounds, since the costs tend to increase with longer chain numbers [92]. Furthermore, as we have illustrated above, even-chain molecules have relatively higher enthalpies of fusion, and they are generally less expensive than odd chains; for example, the *n* = 17 fatty acid (Δ_fus_*H* = 190 J g^−1^) is about 30 times more expensive than the *n* = 18 fatty acid (Δ_fus_*H* = 222 J g^−1^) at 100-gram quantities. In sum, within a given chemical family, it is best to select PCMs with even numbers of carbons, based on the lower quantities required and lower associated material costs, to store a given amount of thermal energy.

### 4.3. Other Practical Matters

Paraffins are odorless, and not considered to be hazardous, but they are flammable, and they have a large volume change (ca. 10%) on melting. A thorough study of paraffin PCMs [27] showed no significant variation in Δ_fus_*H* and *T*_fus_ for paraffins over 3000 melt-freeze cycles, including heating to 90 °C. (The importance of long-term cycling stability for PCMs and experimental devices to quantify this matter have been described recently elsewhere [93]). Furthermore, in that study the paraffins were placed in contact with 17 different materials, including metals, metal alloys, and plastics, at 75 °C for 12 weeks. The results [27] showed excellent compatibility of paraffins with aluminium alloys, stainless steel, most copper, nickel and magnesium alloys and type I PVC, although incompatibilities with polypropylene, cast acrylic, silicone rubber, ABS plastic and nylon. Typically, alkanes show very little crystallization hysteresis, and freeze at temperatures very close to the melting point, although there can be some hysteresis in the solid–solid transitions [94]. As for most organic materials, thermal conductivities of paraffins are low, typically 0.2 to 0.4 W m^−1^ K^−1^ in the solid state, and 0.1 to 0.2 W m^−1^ K^−1^ in the liquid state [27]. 

Fatty acids are minor irritants to the skin, and have flash points. They are generally derived from biosources, and the energy to produce them can be recouped in a matter of months when used for solar thermal storage [95]. A comprehensive investigation of fatty acid PCMs [96] showed stable values of Δ_fus_*H* and *T*_fus_ for fatty acids over 3000 melt-freeze cycles. Even at low purity (<80% pure), the thermal properties of fatty acids are robust [97]. Contact experiments have been carried out for molten fatty acid PCMs at 75 °C and 16 different materials, including aluminum alloys, copper, nickel and magnesium alloys, stainless steel, and various polymers. The molten fatty acids were compatible with aluminum alloys, stainless steel, and polycarbonate, but not with copper, nickel or magnesium alloys, or with polypropylene, cast acrylic, type I PVC, silicone rubber, ABS plastic or nylon [96]. Under most conditions, fatty acids show little supercooling [98], and their volume change on melting is very small [96]. Thermal conductivities of fatty acids are low, typically 0.2 to 0.3 W m^−1^ K^−1^ in the solid state, and 0.1 to 0.2 W m^−1^ K^−1^ in the liquid [96]. Binary eutectics of fatty acids have been investigated and shown to be stable over 3000 melt-freeze cycles [99], and with a wider range of melting points than pure fatty acids [100]. 

Long-chain alcohols are not considered to be hazardous substances. They do have known flash points. Thermal stability of Δ_fus_*H* and *T*_fus_ over 300 cycles is very good for dodecanol [101], and likely similar for other long-chain alcohols. In general terms, these alcohols are relatively unreactive, but detailed studies (as for paraffins and fatty acids) are not available. As described above, the melting transition in long-chain fatty alcohols can be very close to the temperature of solid–solid transitions. The melting point and freezing point can be very close, but the solid–solid transition can be supressed on cooling, for example, for octadecanol, from ca. 56 °C on heating to 50 °C on cooling [96], which could reduce the thermal energy released in some circumstances. Thermal conductivity for dodecanol is ca. 0.2 W m^−1^ K^−1^ in the solid and liquid states [101].

Carboxylic esters can present low toxicity and corrosiveness, and can be biobased [102]. They typically show low supercooling and low volume changes on melting, but are flammable and problematic with plastics [39].

Although the thermal properties of α,ω-disubstituted alkanes can be impressive, as presented above, less is known about their other specific properties. All would be expected to be flammable, and possibly have flash points, and some may decompose on melting. Most diols likely show melting hysteresis, as seen for decane-1,10-diol [103], because polyols require considerable reorganization to establish their multiple hydrogen bonds on crystallization. Dioic acids could be expected to have interactions with other materials similar to what has been observed for fatty acids [96]. Further studies of supercooling, chemical reactivity, volume change on melting, thermal property stability on thousands of melt-freeze cycles and thermal conductivity in both the solid and the liquid phases would be most welcome for α,ω-disubstituted alkanes.

## 5. Conclusions

In this review, we presented trends in melting points (*T*_fus_) and enthalpies of fusion (Δ_fus_*H*) of long-chain unbranched organic molecules with potential applications as phase change materials. The data were extracted from an extensive compilation of nearly 7000 compounds and the analysis focused on the following nine families of materials: alkanes (paraffins), fatty acids, fatty alcohols, esters, diamines, dinitriles, diols, dioic acids, and diamides.

A comparison of the melting points of these nine families showed that the highest melting points, at a given number of carbons in the backbone of the compounds, occur for compounds with strong hydrogen bonding interactions. Diamides exhibit the highest melting points, whereas alkanes and esters have the lowest melting points for a given number of carbons. The general trend within a given family is to higher melting point at higher molecular mass. 

The investigation of the melting points as a function of alkyl chain length, *n*, showed separate trends for even- and odd-carbon chains for both *n*-alkanes and fatty acids. Even-carbon *n*-alkanes and fatty acids generally have higher melting points than odd-carbon chains due to their more efficient packing. Long-chain alcohols also have different packing of the odd and even alkyl chains, but their melting points do not show pronounced odd–even effects. Analysis of the melting points data of esters in terms of the alkyl chain length, *A*, showed that, within a given value of *A*, the melting point increases with the total number of carbons in the compound. For a given number of carbons in the ester, esters with shorter alkyl chains have higher and similar melting points, and esters with longer alkyl chains have lower (and similar) melting points. Odd–even effects also were observed in the melting points of diamines, dinitriles, diols, dioic acids, and diamides, again with the odd chains exhibiting lower melting points, likely due to their less efficient packing.

Analysis of the enthalpies of fusion, Δ_fus_*H*, as a function of chain length showed prominent odd–even chain length effects for *n*-alkanes. Even carbon alkanes with *n* ≤ 20 have much higher values of Δ_fus_*H* than alkanes with odd values of *n*. The difference was attributed to the absence of solid–solid transitions in even carbon alkanes within this range of *n*. For even values of *n* between 22 and 36, there is at least one solid–solid transition and Δ_fus_*H* generally follows the lower Δ_fus_*H* trend of the odd values of *n* at these chain lengths. If the solid–solid transition is within ~5 K of the melting point, its transition enthalpy should likely be included in the energy balance of the latent heat storage system. 

Fatty acids also showed noticeable odd–even effects in Δ_fus_*H* as a function of chain length, and fatty acids with even numbers of carbon exhibited higher values of Δ_fus_*H*. Compared to alkanes with a given chain length, Δ_fus_*H* (in J g^−1^) of a fatty acid with *n* < 20 is a little less than for the corresponding alkane, but for *n* > 20, Δ_fus_*H* of the fatty acids is higher than for the alkanes, and about the same as Δ_tot_*H* for the alkanes. In contrast to alkanes and fatty acids, Δ_fus_*H* of long chain saturated fatty alcohols as a function of chain length showed very subtle odd–even effects. For a given chain length, Δ_fus_*H* (in J g^−1^) of a fatty alcohol is similar to that for the corresponding alkane, although the alcohols have a higher melting point.

For esters, Δ_fus_*H* showed a general trend to higher values as the number of carbons in the molecular structure increased. Most esters investigated in this study have Δ_fus_*H* > 150 J g^−1^ and discernible odd–even effects in Δ_fus_*H* within a given alkyl chain length, *A*. As for alkanes and fatty acids, esters with odd *A* values have lower values of Δ_fus_*H*. For a given number of carbons in the backbone, the general trend is to higher values of Δ_fus_*H* with shorter alkyl chains (smaller *A* values), although these trends are not always observed.

Diamines, dinitriles, diols, dioic acids and diamides all showed odd–even effects, again with higher Δ_fus_*H* values for even carbon numbers. Most remarkable are the high gravimetric Δ_fus_*H* values of these families, especially for diamines and diamides with even numbers of carbon (Δ_fus_*H* > 300 J g^−1^). The relatively strong hydrogen bonding at both ends of these molecules and flexibility of the alkyl chains contribute to these exceptionally high values of Δ_fus_*H.*

Further studies of the properties of diamines, dinitriles, diols, dioic acids and diamines and their suitability as PCMs would be useful. For all potential PCMs, knowledge of supercooling is essential, and DSC traces both on melting and freezing should be presented in published work. For most potential PCMs, investigations of long-term thermal cycling using known methodologies [93] are lacking, and such information would significantly advance the field.

In summary, about 200 long-chain organic molecules were analyzed in this study and trends in their properties are presented in Table 1. The range of their Δ_fus_*H* was 100 to 353 J g^−1^ and their melting points varied from −70 °C to 200 °C, allowing for their use in a wide range of thermal energy storage applications. Use of eutectics could extend the temperature range to even lower temperatures. The data and trends in melting points and Δ_fus_*H* established in this work, along with other considerations, such as availability and cost, chemical compatibility, and thermal and chemical stabilities, can serve as aids in selection of an appropriate PCM from within this extensive range of compounds. For an application requiring a specific transition temperature, compounds from the different families studied here can be chosen. The data show that molecules with even alkyl chains are generally more desirable as they are less expensive and store more thermal energy per unit mass than similar compounds with odd alkyl chains. 

## Figures and Tables

**Table 1:** A summary of the families of long-chain linear organic compounds reviewed in this work. For each family, the ranges of alkyl chain length, *n*, and of the melting points (*T*_fus_) and enthalpies of fusion (Δ_fus_*H*) are indicated. Trends in *T*_fus_ and Δ_fus_*H* observed from this review are also included.

Compounds	*n*	*T*_fus_ (°C)	Trends in *T*_fus_	Δ_fus_*H* (J g^−1^)	Trends in Δ_fus_*H*
*n*-Alkanes (Paraffins)	10–54	−30–95	Increases with *n*. Odd–even effects. Higher for even-carbon.	140–242	Increases with *n*. Higher for even-carbon with *n* ≤ 20.
Alcohols	11–26	16–80	Increases with *n*. Smooth melting curve with almost no odd–even effects.	119–177	Increases with *n*. Subtle odd–even effects.
Fatty acids	11–26	28–80	Increases with *n*. Odd–even effects. Higher for even-carbon.	140–236	Increases with *n*. Odd–even effects. Higher for even-carbon.
Esters	10–38	−74–65	Increases with *n* for a given alkyl length *A*. Higher for shorter alkyl chains.	99–250	Increases with *n* for a given alkyl length *A*. Odd–even effects within a given *A*. Can be higher for shorter alkyl chains.
α,ω-Disubstituted alkanes (diamides, diamines, dinitriles, dioic acids, diols)	8–18	−7–220	Increases with *n*. Odd–even effects. Higher for even-carbon.	146–353	Increases with *n*. Odd–even effects within the same family. Higher for even-carbon.

## Data Availability

All data used in this analysis are presented in the Supporting Information.

## Supplementary Materials

The following supporting information can be downloaded at: https://www.mdpi.com/article/10.3390/molecules26216635/s1. Table S1: Melting points of alkanes with 10 to 55 carbons (data for Figure 1 and Figure 2); Table S2: Thermal properties of alkanes with odd numbers of carbons from 11 to 35 (data for Figure 2 and Figure 5); Table S3: Thermal properties of alkanes with even numbers of carbons from 10 to 46 (data for Figure 2 and Figure 5); Table S4: Melting points and enthalpies of fusion of fatty alcohols with 13 to 26 carbons (data for Figure 1 and Figure 6); Table S5: Melting points and total enthalpies of transition of fatty alcohols with 12 to 26 carbons (data for Figure 6); Table S6: Melting points and enthalpies of fusion of fatty acids with 11 to 26 carbons (data for Figure 1 and Figure 7); Table S7: Melting points and enthalpies of fusion for esters with 10 to 38 carbons (data used for Figure 1, Figure 3 and Figure 8); Table S8: Melting points and enthalpies of fusion of diamides with 8 to 14 carbons (data for Figure 1 and Figure 7); Table S9: Melting points and enthalpies of fusion of diamines with 8 to 12 carbons (data for Figure 1 and Figure 9); Table S10: Melting points and enthalpies of fusion for dinitriles with 10 to 14 carbons (data for Figure 1 and Figure 9); Table S11: Melting points and enthalpies of fusion for diols with 10 to 20 carbons (data for Figure 1 and Figure 9); Table S12: Melting points and enthalpies of fusion of dioic acids with 10 to 18 carbons (data for Figure 1 and Figure 9). All Tables include their literature references.
