# Supplementary material for: Organic Phase Change Materials for Thermal Energy Storage: Influence of Molecular Structure on Properties
Source: Molecules. 2021 Nov 2;26(21):6635. doi: 10.3390/molecules26216635 (PMC8588044; doi:10.3390/molecules26216635)
Supplement: Supplementary file 1 [file molecules-26-06635-s001.zip › molecules-1432623-supplementary.pdf]

**Supplementary Information**

# Organic Phase Change Materials for Thermal Energy Storage: Influence of Molecular Structure on Properties

Samer Kahwaji[a] and Mary Anne White[a,b,*]

[a]Department of Chemistry, Dalhousie University, Halifax, NS B3H 4R2, Canada

[b]Clean Technologies Research Institute, Dalhousie University, Halifax, NS B3H 4R2, Canada

*Author for correspondence; mawhite@dal.ca

**Table S1.** Melting temperatures ($T_{fus}$) of alkanes with carbon number between 10 and 55. These data were used in Figs. 1 and 2 in the manuscript.

| Molecular formula | Molecular weight (g mol$^{-1}$) | Carbon number | $T_{fus}$ (K) | $T_{fus}$ (°C) | References |
|---|---|---|---|---|---|
| $C_{10}H_{22}$ | 142.28 | 10 | 243.50 | -29.65 | Dirand M, Bouroukba M, Briard A-J, Chevallier V, Petitjean D, Corriou J-P. Temperatures and enthalpies of (solid + solid) and (solid + liquid) transitions of n-alkanes. J Chem Thermodyn 2002;34:1255–77. doi:10.1006/jcht.2002.0978. |
| $C_{11}H_{24}$ | 156.31 | 11 | 247.20 | -25.95 | M. J. S. Monte, A. R. R. P. Almeida, and M. A. V. Ribeiro da Silva, J. Chem. Thermodyn. 36, 385 (2004). https://doi.org/10.1016/j.jct.2004.02.001 |
| $C_{11}H_{24}$ | 156.30 | 11 | 247.70 | -25.45 | Dirand M, Bouroukba M, Briard A-J, Chevallier V, Petitjean D, Corriou J-P. Temperatures and enthalpies of (solid + solid) and (solid + liquid) transitions of n-alkanes. J Chem Thermodyn 2002;34:1255–77. doi:10.1006/jcht.2002.0978. |
| $C_{12}H_{26}$ | 170.33 | 12 | 263.50 | -9.65 | D. D. Tunnicliff and H. Stone, Anal. Chem. 27, 73 (1955). https://doi.org/10.1021/ac60097a022 |
| $C_{12}H_{26}$ | 170.34 | 12 | 263.60 | -9.55 | Dirand M, Bouroukba M, Briard A-J, Chevallier V, Petitjean D, Corriou J-P. Temperatures and enthalpies of (solid + solid) and (solid + liquid) transitions of n-alkanes. J Chem Thermodyn 2002;34:1255–77. doi:10.1006/jcht.2002.0978. |
| $C_{13}H_{28}$ | 184.36 | 13 | 267.70 | -5.45 | E. S. Domalski and E. D. Hearing, J. Phys. Chem. Ref. Data 25, 1 (1996), and references therein. https://doi.org/10.1063/1.555985 |
| $C_{13}H_{28}$ | 184.36 | 13 | 267.60 | -5.55 | Dirand M, Bouroukba M, Briard A-J, Chevallier V, Petitjean D, Corriou J-P. Temperatures and enthalpies of (solid + solid) and (solid + liquid) transitions of n-alkanes. J Chem Thermodyn 2002;34:1255–77. doi:10.1006/jcht.2002.0978. |
| $C_{14}H_{30}$ | 198.39 | 14 | 278.30 | 5.15 | M. J. S. Monte, A. R. R. P. Almeida, and M. A. V. Ribeiro da Silva, J. Chem. Thermodyn. 36, 385 (2004). https://doi.org/10.1016/j.jct.2004.02.001 |
| $C_{14}H_{30}$ | 198.39 | 14 | 279.00 | 5.85 | Dirand M, Bouroukba M, Briard A-J, Chevallier V, Petitjean D, Corriou J-P. Temperatures and enthalpies of (solid + solid) and (solid + liquid) transitions of n-alkanes. J Chem Thermodyn 2002;34:1255–77. doi:10.1006/jcht.2002.0978. |
| $C_{15}H_{32}$ | 212.41 | 15 | 282.70 | 9.55 | C. Velez, M. Khayet, and J. M. Ortiz de Zarate, Appl. Energy 143, 383 (2015). https://doi.org/10.1016/j.apenergy.2015.01.054 |
| $C_{15}H_{32}$ | 212.40 | 15 | 283.10 | 9.95 | Dirand M, Bouroukba M, Briard A-J, Chevallier V, Petitjean D, Corriou J-P. Temperatures and enthalpies of (solid + solid) and (solid + liquid) transitions of n-alkanes. J Chem Thermodyn 2002;34:1255–77. doi:10.1006/jcht.2002.0978. |
| $C_{16}H_{34}$ | 226.44 | 16 | 290.90 | 17.75 | M. J. S. Monte, A. R. R. P. Almeida, and M. A. V. Ribeiro da Silva, J. Chem. Thermodyn. 36, 385 (2004). https://doi.org/10.1016/j.jct.2004.02.001 |
| $C_{16}H_{34}$ | 226.44 | 16 | 291.20 | 18.05 | Dirand M, Bouroukba M, Briard A-J, Chevallier V, Petitjean D, Corriou J-P. Temperatures and enthalpies of (solid + solid) and (solid + liquid) transitions of n-alkanes. J Chem Thermodyn 2002;34:1255–77. doi:10.1006/jcht.2002.0978. |
| $C_{17}H_{36}$ | 240.47 | 17 | 294.70 | 21.55 | P. Espeau and J. W. White, Carbon 43, 1885 (2005). https://doi.org/10.1016/j.carbon.2005.02.037 |
| $C_{17}H_{36}$ | 240.47 | 17 | 294.90 | 21.75 | Dirand M, Bouroukba M, Briard A-J, Chevallier V, Petitjean D, Corriou J-P. Temperatures and enthalpies of (solid + solid) and (solid + liquid) transitions of n-alkanes. J Chem Thermodyn 2002;34:1255–77. doi:10.1006/jcht.2002.0978. |
| $C_{18}H_{38}$ | 254.49 | 18 | 300.95 | 27.80 | P. Claudy and J. M. Letoffe, Calorim. Anal. Therm. 22, 281 (1991) |
| $C_{18}H_{38}$ | 254.50 | 18 | 300.20 | 27.05 | Anne-Julie Briard, Mohammed Bouroukba, Dominique Petitjean, Nathalie Hubert and, Dirand* M. Experimental Enthalpy Increments from the Solid Phases to the Liquid Phase of Homologous n-Alkane Series (C18 to C38 and C41, C44, C46, C50, C54, and C60). J Chem Eng Data 2003;48:497–513. doi:10.1021/JE0201368. |
| $C_{19}H_{40}$ | 268.52 | 19 | 305.10 | 31.95 | M. J. S. Monte, A. R. R. P. Almeida, and M. A. V. Ribeiro da Silva, J. Chem. Thermodyn. 36, 385 (2004). https://doi.org/10.1016/j.jct.2004.02.001 |
| $C_{19}H_{40}$ | 268.52 | 19 | 305.00 | 31.85 | Kahwaji S, Johnson MB, Kheirabadi AC, Groulx D, White MA. A comprehensive study of properties of paraffin phase change materials for solar thermal energy storage and thermal management applications. Energy 2018;162:1169–82. doi:10.1016/j.energy.2018.08.068. |
| $C_{20}H_{42}$ | 282.55 | 20 | 309.70 | 36.55 | V. Metivaud, F. Rajabalee, H. A. J. Oonk, D. Mondieig, and Y. Haget, Can. J. Chem. 77, 332 (1999). https://doi.org/10.1139/v99-004 |
| $C_{20}H_{45}$ | 282.55 | 20 | 310.70 | 37.55 | Kahwaji S, Johnson MB, Kheirabadi AC, Groulx D, White MA. A comprehensive study of properties of paraffin phase change materials for solar thermal energy storage and thermal management applications. Energy 2018;162:1169–82. doi:10.1016/j.energy.2018.08.068. |

| Molecular formula | Molecular weight (g mol$^{-1}$) | Carbon number | $T_{fus}$ (K) | $T_{fus}$ (°C) | References |
|---|---|---|---|---|---|
| $C_{21}H_{44}$ | 296.57 | 21 | 313.00 | 39.85 | M. J. S. Monte, A. R. R. P. Almeida, and M. A. V. Ribeiro da Silva, J. Chem. Thermodyn. 36, 385 (2004). https://doi.org/10.1016/j.jct.2004.02.001 |
| $C_{21}H_{49}$ | 296.58 | 21 | 312.00 | 38.85 | Anne-Julie Briard, Mohammed Bouroukba, Dominique Petitjean, Nathalie Hubert and, Dirand* M. Experimental Enthalpy Increments from the Solid Phases to the Liquid Phase of Homologous n-Alkane Series (C18 to C38 and C41, C44, C46, C50, C54, and C60). J Chem Eng Data 2003;48:497–513. doi:10.1021/JE0201368. |
| $C_{22}H_{46}$ | 310.60 | 22 | 316.10 | 42.95 | M. J. S. Monte, A. R. R. P. Almeida, and M. A. V. Ribeiro da Silva, J. Chem. Thermodyn. 36, 385 (2004). https://doi.org/10.1016/j.jct.2004.02.001 |
| $C_{22}H_{46}$ | 310.60 | 22 | 316.95 | 43.80 | Kahwaji S, Johnson MB, Kheirabadi AC, Groulx D, White MA. A comprehensive study of properties of paraffin phase change materials for solar thermal energy storage and thermal management applications. Energy 2018;162:1169–82. doi:10.1016/j.energy.2018.08.068. |
| $C_{23}H_{48}$ | 324.63 | 23 | 320.20 | 47.05 | L. Robles, D. Mondieig, Y. Haget, and M. A. Cuevas-Diarte, J. Chim. Phys. Phys.-Chim. Biol. 95, 92–111 (1998). https://doi.org/10.1051/jcp:1998111 |
| $C_{23}H_{48}$ | 324.63 | 23 | 319.90 | 46.75 | Anne-Julie Briard, Mohammed Bouroukba, Dominique Petitjean, Nathalie Hubert and, Dirand* M. Experimental Enthalpy Increments from the Solid Phases to the Liquid Phase of Homologous n-Alkane Series (C18 to C38 and C41, C44, C46, C50, C54, and C60). J Chem Eng Data 2003;48:497–513. doi:10.1021/JE0201368. |
| $C_{24}H_{50}$ | 338.65 | 24 | 323.80 | 50.65 | E. P. Gilbert, Phys. Chem. Chem. Phys. 1, 1517 (1999). https://doi.org/10.1039/a808664h |
| $C_{24}H_{50}$ | 338.66 | 24 | 323.00 | 49.85 | Anne-Julie Briard, Mohammed Bouroukba, Dominique Petitjean, Nathalie Hubert and, Dirand* M. Experimental Enthalpy Increments from the Solid Phases to the Liquid Phase of Homologous n-Alkane Series (C18 to C38 and C41, C44, C46, C50, C54, and C60). J Chem Eng Data 2003;48:497–513. doi:10.1021/JE0201368. |
| $C_{25}H_{52}$ | 352.68 | 25 | 326.70 | 53.55 | E. S. Domalski and E. D. Hearing, J. Phys. Chem. Ref. Data 25, 1 (1996), and references therein. https://doi.org/10.1063/1.555985 |
| $C_{25}H_{52}$ | 352.69 | 25 | 327.40 | 54.25 | Anne-Julie Briard, Mohammed Bouroukba, Dominique Petitjean, Nathalie Hubert and, Dirand* M. Experimental Enthalpy Increments from the Solid Phases to the Liquid Phase of Homologous n-Alkane Series (C18 to C38 and C41, C44, C46, C50, C54, and C60). J Chem Eng Data 2003;48:497–513. doi:10.1021/JE0201368. |
| $C_{26}H_{54}$ | 366.71 | 26 | 329.60 | 56.45 | U. Domanska and D. Wyrzykowska-Starkiewicz, Thermochim. Acta 179, 265 (1991). https://doi.org/10.1016/0040-6031(91)80356-N |
| $C_{26}H_{52}$ | 366.71 | 26 | 328.90 | 55.75 | Anne-Julie Briard, Mohammed Bouroukba, Dominique Petitjean, Nathalie Hubert and, Dirand* M. Experimental Enthalpy Increments from the Solid Phases to the Liquid Phase of Homologous n-Alkane Series (C18 to C38 and C41, C44, C46, C50, C54, and C60). J Chem Eng Data 2003;48:497–513. doi:10.1021/JE0201368. |
| $C_{27}H_{56}$ | 380.73 | 27 | 332.00 | 58.85 | E. P. Gilbert, Phys. Chem. Chem. Phys. 1, 1517 (1999). https://doi.org/10.1039/a808664h |
| $C_{27}H_{56}$ | 380.74 | 27 | 331.20 | 58.05 | Anne-Julie Briard, Mohammed Bouroukba, Dominique Petitjean, Nathalie Hubert and, Dirand* M. Experimental Enthalpy Increments from the Solid Phases to the Liquid Phase of Homologous n-Alkane Series (C18 to C38 and C41, C44, C46, C50, C54, and C60). J Chem Eng Data 2003;48:497–513. doi:10.1021/JE0201368. |
| $C_{28}H_{58}$ | 394.76 | 28 | 334.50 | 61.35 | I. Paunovic and A. K. Mehrotra, Thermochim. Acta 356, 27 (2000). https://doi.org/10.1016/S0040-6031(00)00503-7 |
| $C_{28}H_{59}$ | 394.77 | 28 | 333.20 | 60.05 | Anne-Julie Briard, Mohammed Bouroukba, Dominique Petitjean, Nathalie Hubert and, Dirand* M. Experimental Enthalpy Increments from the Solid Phases to the Liquid Phase of Homologous n-Alkane Series (C18 to C38 and C41, C44, C46, C50, C54, and C60). J Chem Eng Data 2003;48:497–513. doi:10.1021/JE0201368. |
| $C_{29}H_{60}$ | 408.79 | 29 | 336.80 | 63.65 | E. P. Gilbert, Phys. Chem. Chem. Phys. 1, 1517 (1999). https://doi.org/10.1039/a808664h |
| $C_{29}H_{60}$ | 409.79 | 29 | 336.00 | 62.85 | Anne-Julie Briard, Mohammed Bouroukba, Dominique Petitjean, Nathalie Hubert and, Dirand* M. Experimental Enthalpy Increments from the Solid Phases to the Liquid Phase of Homologous n-Alkane Series (C18 to C38 and C41, C44, C46, C50, C54, and C60). J Chem Eng Data 2003;48:497–513. doi:10.1021/JE0201368. |
| $C_{30}H_{62}$ | 422.81 | 30 | 338.70 | 65.55 | N. V. Platonova, E. N. Kotel'nikova, S. K. Filatov, G. A. Puchkovskaya, I. I. Gnatyuk, J. Baran, and M. Drozd, J. Struct. Chem. 53, 973 (2012). https://doi.org/10.1134/S0022476612050228 |
| $C_{30}H_{62}$ | 422.82 | 30 | 337.50 | 64.35 | Anne-Julie Briard, Mohammed Bouroukba, Dominique Petitjean, Nathalie Hubert and, Dirand* M. Experimental Enthalpy Increments from the Solid Phases to the Liquid Phase of Homologous n-Alkane Series (C18 to C38 and C41, C44, C46, C50, C54, and C60). J Chem Eng Data 2003;48:497–513. doi:10.1021/JE0201368. |

| Molecular formula | Molecular weight (g mol$^{-1}$) | Carbon number | $T_{fus}$ (K) | $T_{fus}$ (°C) | References |
|---|---|---|---|---|---|
| $C_{31}H_{64}$ | 436.84 | 31 | 341.10 | 67.95 | I. Boudouh, I. Djemai, J. A. Gonzalez, and D. Barkat, J. Mol. Liq. 216, 764 (2016). https://doi.org/10.1016/j.molliq.2016.02.010 |
| $C_{31}H_{64}$ | 436.85 | 31 | 340.00 | 66.85 | Anne-Julie Briard, Mohammed Bouroukba, Dominique Petitjean, Nathalie Hubert and, Dirand* M. Experimental Enthalpy Increments from the Solid Phases to the Liquid Phase of Homologous n-Alkane Series (C18 to C38 and C41, C44, C46, C50, C54, and C60). J Chem Eng Data 2003;48:497–513. doi:10.1021/JE0201368. |
| $C_{32}H_{66}$ | 450.87 | 32 | 343.50 | 70.35 | E. P. Gilbert, Phys. Chem. Chem. Phys. 1, 1517 (1999). https://doi.org/10.1039/a808664h |
| $C_{32}H_{66}$ | 450.87 | 32 | 341.90 | 68.75 | Anne-Julie Briard, Mohammed Bouroukba, Dominique Petitjean, Nathalie Hubert and, Dirand* M. Experimental Enthalpy Increments from the Solid Phases to the Liquid Phase of Homologous n-Alkane Series (C18 to C38 and C41, C44, C46, C50, C54, and C60). J Chem Eng Data 2003;48:497–513. doi:10.1021/JE0201368. |
| $C_{33}H_{68}$ | 464.89 | 33 | 344.80 | 71.65 | E. P. Gilbert, Phys. Chem. Chem. Phys. 1, 1517 (1999). https://doi.org/10.1039/a808664h |
| $C_{33}H_{68}$ | 464.90 | 33 | 343.40 | 70.25 | Anne-Julie Briard, Mohammed Bouroukba, Dominique Petitjean, Nathalie Hubert and, Dirand* M. Experimental Enthalpy Increments from the Solid Phases to the Liquid Phase of Homologous n-Alkane Series (C18 to C38 and C41, C44, C46, C50, C54, and C60). J Chem Eng Data 2003;48:497–513. doi:10.1021/JE0201368. |
| $C_{34}H_{70}$ | 478.92 | 34 | 346.00 | 72.85 | L. Ventola, M. A. Cuevas-Diarte, T. Calvet, I. Angulo, M. Vivanco, M. Bernar, G. Bernar, M. Melero, and D. Mondieig, J. Phys. Chem. Solids 66, 1668 (2005). https://doi.org/10.1016/j.jpcs.2005.06.001 |
| $C_{34}H_{70}$ | 478.93 | 34 | 345.30 | 72.15 | Anne-Julie Briard, Mohammed Bouroukba, Dominique Petitjean, Nathalie Hubert and, Dirand* M. Experimental Enthalpy Increments from the Solid Phases to the Liquid Phase of Homologous n-Alkane Series (C18 to C38 and C41, C44, C46, C50, C54, and C60). J Chem Eng Data 2003;48:497–513. doi:10.1021/JE0201368. |
| $C_{35}H_{72}$ | 492.95 | 35 | 347.20 | 74.05 | H. Yamamoto, S. Teshima, N. Nemoto, and K. Tashiro, J. Phys. Chem. A 113, 2632 (2009). https://doi.org/10.1021/jp808176n |
| $C_{35}H_{72}$ | 492.95 | 35 | 346.50 | 73.35 | Anne-Julie Briard, Mohammed Bouroukba, Dominique Petitjean, Nathalie Hubert and, Dirand* M. Experimental Enthalpy Increments from the Solid Phases to the Liquid Phase of Homologous n-Alkane Series (C18 to C38 and C41, C44, C46, C50, C54, and C60). J Chem Eng Data 2003;48:497–513. doi:10.1021/JE0201368. |
| $C_{36}H_{74}$ | 506.97 | 36 | 348.90 | 75.75 | L. Ventola, M. A. Cuevas-Diarte, T. Calvet, I. Angulo, M. Vivanco, M. Bernar, G. Bernar, M. Melero, and D. Mondieig, J. Phys. Chem. Solids 66, 1668 (2005). https://doi.org/10.1016/j.jpcs.2005.06.001 |
| $C_{36}H_{74}$ | 506.98 | 36 | 348.90 | 75.75 | Anne-Julie Briard, Mohammed Bouroukba, Dominique Petitjean, Nathalie Hubert and, Dirand* M. Experimental Enthalpy Increments from the Solid Phases to the Liquid Phase of Homologous n-Alkane Series (C18 to C38 and C41, C44, C46, C50, C54, and C60). J Chem Eng Data 2003;48:497–513. doi:10.1021/JE0201368. |
| $C_{40}H_{82}$ | 563.08 | 40 | 353.20 | 80.05 | L. Ventola, M. A. Cuevas-Diarte, T. Calvet, I. Angulo, M. Vivanco, M. Bernar, G. Bernar, M. Melero, and D. Mondieig, J. Phys. Chem. Solids 66, 1668 (2005). https://doi.org/10.1016/j.jpcs.2005.06.001 |
| $C_{40}H_{82}$ | 563.08 | 40 | 354.00 | 80.85 | Dirand M, Bouroukba M, Briard A-J, Chevallier V, Petitjean D, Corriou J-P. Temperatures and enthalpies of (solid + solid) and (solid + liquid) transitions of n-alkanes. J Chem Thermodyn 2002;34:1255–77. doi:10.1006/jcht.2002.0978. |
| $C_{41}H_{84}$ | 577.11 | 41 | 354.30 | 81.15 | A.-J. Briard, M. Bouroukba, D. Petitjean, N. Hubert, and M. Dirand, J. Chem. Eng. Data 48, 497 (2003). https://doi.org/10.1021/je0201368 |
| $C_{42}H_{86}$ | 591.13 | 42 | 357.30 | 84.15 | M.-H. Wang, Z.-C. Tan, Q. Shi, L.-X. Sun, and T. Zhang, J. Therm. Anal. Calorim. 84, 413 (2006). https://doi.org/10.1007/s10973-005-6971-6 |
| $C_{44}H_{90}$ | 619.19 | 44 | 358.50 | 85.35 | L. Ventola, M. A. Cuevas-Diarte, T. Calvet, I. Angulo, M. Vivanco, M. Bernar, G. Bernar, M. Melero, and D. Mondieig, J. Phys. Chem. Solids 66, 1668 (2005). https://doi.org/10.1016/j.jpcs.2005.06.001 |
| $C_{44}H_{90}$ | 619.19 | 44 | 358.75 | 85.60 | Dirand M, Bouroukba M, Briard A-J, Chevallier V, Petitjean D, Corriou J-P. Temperatures and enthalpies of (solid + solid) and (solid + liquid) transitions of n-alkanes. J Chem Thermodyn 2002;34:1255–77. doi:10.1006/jcht.2002.0978. |
| $C_{46}H_{94}$ | 647.24 | 46 | 360.70 | 87.55 | A.-J. Briard, M. Bouroukba, D. Petitjean, N. Hubert, and M. Dirand, J. Chem. Eng. Data 48, 497 (2003). https://doi.org/10.1021/je0201368 |
| $C_{46}H_{94}$ | 647.24 | 46 | 360.70 | 87.55 | Dirand M, Bouroukba M, Briard A-J, Chevallier V, Petitjean D, Corriou J-P. Temperatures and enthalpies of (solid + solid) and (solid + liquid) transitions of n-alkanes. J Chem Thermodyn 2002;34:1255–77. doi:10.1006/jcht.2002.0978. |
| $C_{50}H_{102}$ | 703.34 | 50 | 365.60 | 92.45 | M. J. Hampden Smith and T. T. Kodas, Chem. Vap. Deposition 1, 8 (1995). https://doi.org/10.1002/cvde.19950010103 |
| $C_{50}H_{102}$ | 703.34 | 50 | 365.25 | 92.10 | Dirand M, Bouroukba M, Briard A-J, Chevallier V, Petitjean D, Corriou J-P. Temperatures and enthalpies of (solid + solid) and (solid + liquid) transitions of n-alkanes. J Chem Thermodyn 2002;34:1255–77. doi:10.1006/jcht.2002.0978. |

| Molecular formula | Molecular weight (g mol$^{-1}$) | Carbon number | $T_{fus}$ (K) | $T_{fus}$ (°C) | References |
|---|---|---|---|---|---|
| $C_{52}H_{106}$ | 731.40 | 52 | 366.70 | 93.55 | K. Takamizawa, Y. Urabe, J. Fujimoto, H. Ogata, and Y. Ogawa, Thermochim. Acta 267, 297 (1995). https://doi.org/10.1016/0040-6031(95)02487-5 |
| $C_{54}H_{110}$ | 759.45 | 54 | 368.00 | 94.85 | A.-J. Briard, M. Bouroukba, D. Petitjean, N. Hubert, and M. Dirand, J. Chem. Eng. Data 48, 497 (2003). https://doi.org/10.1021/je0201368 |

**Table S2.** Thermal properties of alkanes with odd number of carbons. The solid-solid transition temperatures ($T_{ss}$) and temperatures of fusion ($T_{fus}$) are listed along with the enthalpies of fusion ($\Delta_{fus}H$) and the total enthalpies of transition ($\Delta_{tot}H = \Delta_{fus}H + \Delta_{ss}H$). These data were used in Figs. 2 and 5 in the manuscript.

| Molecular formula | Molecular weight (g mol$^{-1}$) | Carbon number | $T_{ss}$ (K) | $T_{ss}$ (°C) | $T_{fus}$ (K) | $T_{fus}$ (°C) | $\Delta_{fus}H$ (J g$^{-1}$) | $\Delta_{tot}H$ (J g$^{-1}$) | References |
|---|---|---|---|---|---|---|---|---|---|
| $C_{11}H_{24}$ | 156.30 | 11 | - | - | 247.70 | -25.45 | 142.20 | 186.10 | Dirand M, Bouroukba M, Briard A-J, Chevallier V, Petitjean D, Corriou J-P. Temperatures and enthalpies of (solid + solid) and (solid + liquid) transitions of n-alkanes. J Chem Thermodyn 2002;34:1255–77. doi:10.1006/jcht.2002.0978. |
| $C_{13}H_{28}$ | 184.36 | 13 | 255.00 | -18.15 | 267.60 | -5.55 | 154.50 | 196.00 | Dirand M, Bouroukba M, Briard A-J, Chevallier V, Petitjean D, Corriou J-P. Temperatures and enthalpies of (solid + solid) and (solid + liquid) transitions of n-alkanes. J Chem Thermodyn 2002;34:1255–77. doi:10.1006/jcht.2002.0978. |
| $C_{15}H_{32}$ | 212.40 | 15 | 270.90 | -2.25 | 283.10 | 9.95 | 162.80 | 205.90 | Dirand M, Bouroukba M, Briard A-J, Chevallier V, Petitjean D, Corriou J-P. Temperatures and enthalpies of (solid + solid) and (solid + liquid) transitions of n-alkanes. J Chem Thermodyn 2002;34:1255–77. doi:10.1006/jcht.2002.0978. |
| $C_{17}H_{36}$ | 240.47 | 17 | 283.80 | 10.65 | 294.90 | 21.75 | 166.90 | 212.40 | Dirand M, Bouroukba M, Briard A-J, Chevallier V, Petitjean D, Corriou J-P. Temperatures and enthalpies of (solid + solid) and (solid + liquid) transitions of n-alkanes. J Chem Thermodyn 2002;34:1255–77. doi:10.1006/jcht.2002.0978. |
| $C_{19}H_{40}$ | 268.52 | 19 | 295.00 | 21.85 | 305.00 | 31.85 | 181.00 | 215.00 | Kahwaji S, Johnson MB, Kheirabadi AC, Groulx D, White MA. A comprehensive study of properties of paraffin phase change materials for solar thermal energy storage and thermal management applications. Energy 2018;162:1169–82. doi:10.1016/j.energy.2018.08.068. |
| $C_{21}H_{44}$ | 296.58 | 21 | 304.40 | 31.25 | 312.00 | 38.85 | 160.16 | 216.13 | Anne-Julie Briard, Mohammed Bouroukba, Dominique Petitjean, Nathalie Hubert and, Dirand* M. Experimental Enthalpy Increments from the Solid Phases to the Liquid Phase of Homologous n-Alkane Series (C18 to C38 and C41, C44, C46, C50, C54, and C60). J Chem Eng Data 2003;48:497–513. doi:10.1021/JE0201368. |
| $C_{23}H_{48}$ | 324.63 | 23 | 312.80 | 39.65 | 319.90 | 46.75 | 162.95 | 231.96 | Anne-Julie Briard, Mohammed Bouroukba, Dominique Petitjean, Nathalie Hubert and, Dirand* M. Experimental Enthalpy Increments from the Solid Phases to the Liquid Phase of Homologous n-Alkane Series (C18 to C38 and C41, C44, C46, C50, C54, and C60). J Chem Eng Data 2003;48:497–513. doi:10.1021/JE0201368. |
| $C_{25}H_{52}$ | 352.69 | 25 | 318.80 | 45.65 | 327.40 | 54.25 | 166.44 | 247.53 | Anne-Julie Briard, Mohammed Bouroukba, Dominique Petitjean, Nathalie Hubert and, Dirand* M. Experimental Enthalpy Increments from the Solid Phases to the Liquid Phase of Homologous n-Alkane Series (C18 to C38 and C41, C44, C46, C50, C54, and C60). J Chem Eng Data 2003;48:497–513. doi:10.1021/JE0201368. |

| Molecular formula | Molecular weight (g mol$^{-1}$) | Carbon number | $T_{ss}$ (K) | $T_{ss}$ (°C) | $T_{fus}$ (K) | $T_{fus}$ (°C) | $\Delta_{fus}H$ (J g$^{-1}$) | $\Delta_{tot}H$ (J g$^{-1}$) | References |
|---|---|---|---|---|---|---|---|---|---|
| $C_{27}H_{56}$ | 380.74 | 27 | 325.00 | 51.85 | 331.20 | 58.05 | 180.96 | 258.44 | Anne-Julie Briard, Mohammed Bouroukba, Dominique Petitjean, Nathalie Hubert and, Dirand* M. Experimental Enthalpy Increments from the Solid Phases to the Liquid Phase of Homologous n-Alkane Series (C18 to C38 and C41, C44, C46, C50, C54, and C60). J Chem Eng Data 2003;48:497–513. doi:10.1021/JE0201368. |
| $C_{29}H_{60}$ | 409.79 | 29 | 330.30 | 57.15 | 336.00 | 62.85 | 165.45 | 245.98 | Anne-Julie Briard, Mohammed Bouroukba, Dominique Petitjean, Nathalie Hubert and, Dirand* M. Experimental Enthalpy Increments from the Solid Phases to the Liquid Phase of Homologous n-Alkane Series (C18 to C38 and C41, C44, C46, C50, C54, and C60). J Chem Eng Data 2003;48:497–513. doi:10.1021/JE0201368. |
| $C_{31}H_{64}$ | 436.85 | 31 | 335.20 | 62.05 | 340.00 | 66.85 | 167.79 | 251.57 | Anne-Julie Briard, Mohammed Bouroukba, Dominique Petitjean, Nathalie Hubert and, Dirand* M. Experimental Enthalpy Increments from the Solid Phases to the Liquid Phase of Homologous n-Alkane Series (C18 to C38 and C41, C44, C46, C50, C54, and C60). J Chem Eng Data 2003;48:497–513. doi:10.1021/JE0201368. |
| $C_{33}H_{68}$ | 464.90 | 33 | 337.90 | 64.75 | 343.40 | 70.25 | 173.59 | 255.11 | Anne-Julie Briard, Mohammed Bouroukba, Dominique Petitjean, Nathalie Hubert and, Dirand* M. Experimental Enthalpy Increments from the Solid Phases to the Liquid Phase of Homologous n-Alkane Series (C18 to C38 and C41, C44, C46, C50, C54, and C60). J Chem Eng Data 2003;48:497–513. doi:10.1021/JE0201368. |
| $C_{35}H_{72}$ | 492.95 | 35 | 344.20 | 71.05 | 346.50 | 73.35 | 175.27 | 264.73 | Anne-Julie Briard, Mohammed Bouroukba, Dominique Petitjean, Nathalie Hubert and, Dirand* M. Experimental Enthalpy Increments from the Solid Phases to the Liquid Phase of Homologous n-Alkane Series (C18 to C38 and C41, C44, C46, C50, C54, and C60). J Chem Eng Data 2003;48:497–513. doi:10.1021/JE0201368. |

**Table S3.** Thermal properties of alkanes with even number of carbons. The solid-solid transition temperatures ($T_{ss}$) and melting temperatures ($T_{fus}$) are listed along with the enthalpies of fusion ($\Delta_{fus}H$) and the total enthalpies of transition ($\Delta_{tot}H = \Delta_{fus}H + \Delta_{ss}H$), when available from the cited references. These data were used in Figs. 2 and 5 in the manuscript.

| Molecular formula | Molecular weight (g mol$^{-1}$) | Carbon number | $T_{ss}$ (K) | $T_{ss}$ (°C) | $T_{fus}$ (K) | $T_{fus}$ (°C) | $\Delta_{fus}H$ (J g$^{-1}$) | $\Delta_{tot}H$ (J g$^{-1}$) | References |
|---|---|---|---|---|---|---|---|---|---|
| $C_{10}H_{22}$ | 142.28 | 10 | - | - | 243.50 | -29.65 | - | 201.70 | Dirand M, Bouroukba M, Briard A-J, Chevallier V, Petitjean D, Corriou J-P. Temperatures and enthalpies of (solid + solid) and (solid + liquid) transitions of n-alkanes. J Chem Thermodyn 2002;34:1255–77. doi:10.1006/jcht.2002.0978. |
| $C_{12}H_{26}$ | 170.34 | 12 | - | - | 263.60 | -9.55 | - | 215.80 | Dirand M, Bouroukba M, Briard A-J, Chevallier V, Petitjean D, Corriou J-P. Temperatures and enthalpies of (solid + solid) and (solid + liquid) transitions of n-alkanes. J Chem Thermodyn 2002;34:1255–77. doi:10.1006/jcht.2002.0978. |
| $C_{14}H_{30}$ | 198.39 | 14 | - | - | 279.00 | 5.85 | - | 227.00 | Dirand M, Bouroukba M, Briard A-J, Chevallier V, Petitjean D, Corriou J-P. Temperatures and enthalpies of (solid + solid) and (solid + liquid) transitions of n-alkanes. J Chem Thermodyn 2002;34:1255–77. doi:10.1006/jcht.2002.0978. |
| $C_{16}H_{34}$ | 226.44 | 16 | - | - | 291.20 | 18.05 | - | 235.50 | Dirand M, Bouroukba M, Briard A-J, Chevallier V, Petitjean D, Corriou J-P. Temperatures and enthalpies of (solid + solid) and (solid + liquid) transitions of n-alkanes. J Chem Thermodyn 2002;34:1255–77. doi:10.1006/jcht.2002.0978. |
| $C_{18}H_{38}$ | 254.5 | 18 | - | - | 300.20 | 27.05 | - | 241.65 | Anne-Julie Briard, Mohammed Bouroukba, Dominique Petitjean, Nathalie Hubert and, Dirand* M. Experimental Enthalpy Increments from the Solid Phases to the Liquid Phase of Homologous n-Alkane Series (C18 to C38 and C41, C44, C46, C50, C54, and C60). J Chem Eng Data 2003;48:497–513. doi:10.1021/JE0201368. |
| $C_{20}H_{45}$ | 282.55 | 20 | - | - | 310.70 | 37.55 | - | 246.00 | Kahwaji S, Johnson MB, Kheirabadi AC, Groulx D, White MA. A comprehensive study of properties of paraffin phase change materials for solar thermal energy storage and thermal management applications. Energy 2018;162:1169–82. doi:10.1016/j.energy.2018.08.068. |
| $C_{22}H_{46}$ | 310.60 | 22 | 315.00 | -41.85 | 316.95 | 43.80 | 140.40 | 234.00 | Kahwaji S, Johnson MB, Kheirabadi AC, Groulx D, White MA. A comprehensive study of properties of paraffin phase change materials for solar thermal energy storage and thermal management applications. Energy 2018;162:1169–82. doi:10.1016/j.energy.2018.08.068. |
| $C_{24}H_{50}$ | 338.66 | 24 | 319.90 | -46.75 | 323.00 | 49.85 | 168.90 | 257.19 | Anne-Julie Briard, Mohammed Bouroukba, Dominique Petitjean, Nathalie Hubert and, Dirand* M. Experimental Enthalpy Increments from the Solid Phases to the Liquid Phase of Homologous n-Alkane Series (C18 to C38 and C41, C44, C46, C50, C54, and C60). J Chem Eng Data 2003;48:497–513. doi:10.1021/JE0201368. |
| $C_{26}H_{52}$ | 366.71 | 26 | 325.00 | -51.85 | 328.90 | 55.75 | 167.43 | 266.97 | Anne-Julie Briard, Mohammed Bouroukba, Dominique Petitjean, Nathalie Hubert and, Dirand* M. Experimental Enthalpy Increments from the Solid Phases to the Liquid Phase of Homologous n-Alkane Series (C18 to C38 and C41, C44, C46, C50, C54, and C60). J Chem Eng Data 2003;48:497–513. doi:10.1021/JE0201368. |
| $C_{28}H_{59}$ | 394.77 | 28 | 330.10 | -56.95 | 333.20 | 60.05 | 160.85 | 254.33 | Anne-Julie Briard, Mohammed Bouroukba, Dominique Petitjean, Nathalie Hubert and, Dirand* M. Experimental Enthalpy Increments from the Solid Phases to the Liquid Phase of Homologous n-Alkane Series (C18 to C38 and C41, C44, C46, C50, C54, and C60). J Chem Eng Data 2003;48:497–513. doi:10.1021/JE0201368. |
| $C_{30}H_{62}$ | 422.82 | 30 | 332.10 | -58.95 | 337.50 | 64.35 | 165.08 | 261.58 | Anne-Julie Briard, Mohammed Bouroukba, Dominique Petitjean, Nathalie Hubert and, Dirand* M. Experimental Enthalpy Increments from the Solid Phases to the Liquid Phase of Homologous n-Alkane Series (C18 to C38 and C41, C44, C46, C50, C54, and C60). J Chem Eng Data 2003;48:497–513. doi:10.1021/JE0201368. |

| Molecular formula | Molecular weight (g mol$^{-1}$) | Carbon number | $T_{ss}$ (K) | $T_{ss}$ (°C) | $T_{fus}$ (K) | $T_{fus}$ (°C) | $\Delta_{fus}H$ (J g$^{-1}$) | $\Delta_{tot}H$ (J g$^{-1}$) | References |
|---|---|---|---|---|---|---|---|---|---|
| $C_{32}H_{66}$ | 450.87 | 32 | 338.00 | -64.85 | 341.90 | 68.75 | 170.78 | 268.81 | Anne-Julie Briard, Mohammed Bouroukba, Dominique Petitjean, Nathalie Hubert and, Dirand* M. Experimental Enthalpy Increments from the Solid Phases to the Liquid Phase of Homologous n-Alkane Series (C18 to C38 and C41, C44, C46, C50, C54, and C60). J Chem Eng Data 2003;48:497–513. doi:10.1021/JE0201368. |
| $C_{34}H_{70}$ | 478.93 | 34 | 341.10 | -67.95 | 345.30 | 72.15 | 176.43 | 271.23 | Anne-Julie Briard, Mohammed Bouroukba, Dominique Petitjean, Nathalie Hubert and, Dirand* M. Experimental Enthalpy Increments from the Solid Phases to the Liquid Phase of Homologous n-Alkane Series (C18 to C38 and C41, C44, C46, C50, C54, and C60). J Chem Eng Data 2003;48:497–513. doi:10.1021/JE0201368. |
| $C_{36}H_{74}$ | 506.98 | 36 | 346.10 | -72.95 | 348.90 | 75.75 | 172.99 | 234.33 | Anne-Julie Briard, Mohammed Bouroukba, Dominique Petitjean, Nathalie Hubert and, Dirand* M. Experimental Enthalpy Increments from the Solid Phases to the Liquid Phase of Homologous n-Alkane Series (C18 to C38 and C41, C44, C46, C50, C54, and C60). J Chem Eng Data 2003;48:497–513. doi:10.1021/JE0201368. |
| $C_{40}H_{82}$ | 563.08 | 40 | 353.50 | -80.35 | 354.00 | 80.85 | 240.70 | 265.60 | Dirand M, Bouroukba M, Briard A-J, Chevallier V, Petitjean D, Corriou J-P. Temperatures and enthalpies of (solid + solid) and (solid + liquid) transitions of n-alkanes. J Chem Thermodyn 2002;34:1255–77. doi:10.1006/jcht.2002.0978. |
| $C_{44}H_{90}$ | 619.19 | 44 | 357.70 | -84.55 | 358.75 | 85.60 | 241.70 | 271.10 | Dirand M, Bouroukba M, Briard A-J, Chevallier V, Petitjean D, Corriou J-P. Temperatures and enthalpies of (solid + solid) and (solid + liquid) transitions of n-alkanes. J Chem Thermodyn 2002;34:1255–77. doi:10.1006/jcht.2002.0978. |
| $C_{46}H_{94}$ | 647.24 | 46 | - | 273.15 | 360.70 | 87.55 | - | 272.00 | Dirand M, Bouroukba M, Briard A-J, Chevallier V, Petitjean D, Corriou J-P. Temperatures and enthalpies of (solid + solid) and (solid + liquid) transitions of n-alkanes. J Chem Thermodyn 2002;34:1255–77. doi:10.1006/jcht.2002.0978. |

**Table S4**. Melting temperatures ($T_{fus}$) and enthalpies of fusion ($\Delta_{fus}H$) of fatty alcohols with carbon numbers between 13 and 26. For compounds with more than one data source, the average value of $\Delta_{fus}H$ was calculated. These data were used Figs.1 and 6 in the manuscript.

| Molecular formula | Molecular weight (g mol$^{-1}$) | Carbon number | Compound name | $T_{fus}$ (K) | $T_{fus}$ (°C) | $\Delta_{fus}H$ (J g$^{-1}$) | Average $\Delta_{fus}H$ (J g$^{-1}$) | References |
|---|---|---|---|---|---|---|---|---|
| $C_{13}H_{28}O$ | 200.361 | 13 | 1-tridecanol | 303.500 | 30.350 | 116.29 | 120.21 | J. C. Van Miltenburg, G. J. K. van den Berg, and M. Ramirez, J. Chem. Eng. Data 48, 36 (2003). https://doi.org/10.1021/je025524o |
| $C_{13}H_{28}O$ | 200.361 | 13 | | 318.300 | 45.150 | 124.13 | | V. M. Egorov, V. A. Marikhin, and L. P. Myasnikova, Phys. Solid State 50, 126 (2008). https://doi.org/10.1134/S106378340801023X |
| $C_{14}H_{30}O$ | 214.387 | 14 | 1-tetradecanol | 310.800 | 37.650 | 117.08 | 118.94 | T. M. T. Carvalho, L. M. P. F. Amaral, V. M. F. Morais, and M. D. M. C. Ribeiro da Silva, J. Chem. Thermodyn. 85, 129 (2015). https://doi.org/10.1016/j.jct.2015.01.012 |
| $C_{14}H_{30}O$ | 214.387 | 14 | | 311.200 | 38.050 | 120.81 | | N. D. D. Carareto, T. Castagnaro, M. C. Costa, and A. J. A. Meirelles, J. Chem. Thermodyn. 78, 99 (2014). https://doi.org/10.1016/j.jct.2014.06.011 |
| $C_{15}H_{32}O$ | 228.414 | 15 | 1-pentadecanol | 316.400 | 43.250 | 129.59 | 131.23 | L. Ventola, T. Calvet, M. A. Cuevas-Diarte, M. Ramirez, H. A. J. Oonk, D. Mondieig, and P. Negrier, Phys. Chem. Chem. Phys. 6, 1786 (2004). https://doi.org/10.1039/B313106H |
| $C_{15}H_{32}O$ | 228.414 | 15 | | 316.900 | 43.750 | 132.87 | | J. C. Van Miltenburg, G. J. K. van den Berg, and M. Ramirez, J. Chem. Eng. Data 48, 36 (2003). https://doi.org/10.1021/je025524o |
| $C_{16}H_{34}O$ | 242.441 | 16 | 1-hexadecanol | 321.600 | 48.450 | 136.53 | 138.48 | N. D. D. Carareto, T. Castagnaro, M. C. Costa, and A. J. A. Meirelles, J. Chem. Thermodyn. 78, 99 (2014). https://doi.org/10.1016/j.jct.2014.06.011 |
| $C_{16}H_{34}O$ | 242.441 | 16 | | 322.000 | 48.850 | 127.04 | | T. M. T. Carvalho, L. M. P. F. Amaral, V. M. F. Morais, and M. D. M. C. Ribeiro da Silva, J. Chem. Thermodyn. 97, 70 (2016). https://doi.org/10.1016/j.jct.2016.01.006 |
| $C_{16}H_{34}O$ | 242.441 | 16 | | 322.300 | 49.150 | 138.59 | | J. Xing, Z.-C. Tan, Q. Shi, B. Tong, S.-X. Wang, and Y.-S. Li, J. Therm. Anal. Calorim. 92, 375 (2008). https://doi.org/10.1007/s10973-007-8955-1 |
| $C_{16}H_{34}O$ | 242.441 | 16 | | 322.900 | 49.750 | 140.12 | | V. Metivaud, A. Lefevre, L. Ventola, P. Negrier, E. Moreno, T. Calvet, D. Mondieig, and M. A. Cuevas-Diarte, Chem. Mater. 17, 3302 (2005). https://doi.org/10.1021/cm050130c |
| $C_{16}H_{34}O$ | 242.441 | 16 | | 323.300 | 50.150 | 150.14 | | L. Ventola, T. Calvet, M. A. Cuevas-Diarte, M. Ramirez, H. A. J. Oonk, D. Mondieig, and P. Negrier, Phys. Chem. Chem. Phys. 6, 1786 (2004). https://doi.org/10.1039/B313106H |
| $C_{17}H_{36}O$ | 256.467 | 17 | 1-heptadecanol | 326.600 | 53.450 | 144.27 | 144.27 | J. C. Van Miltenburg, G. J. K. van den Berg, and M. Ramirez, J. Chem. Eng. Data 48, 36 (2003). https://doi.org/10.1021/je025524o |
| $C_{18}H_{38}O$ | 270.494 | 18 | 1-octadecanol | 330.100 | 56.950 | 148.25 | 148.62 | N. D. D. Carareto, T. Castagnaro, M. C. Costa, and A. J. A. Meirelles, J. Chem. Thermodyn. 78, 99 (2014). https://doi.org/10.1016/j.jct.2014.06.011 |
| $C_{18}H_{38}O$ | 270.494 | 18 | | 331.600 | 58.450 | 148.99 | | G. J. Maximo, N. D. D. Carereto, M. C. Costa, A. O. dos Santos, L. P. Cardoso, M. A. Krahenbuhl, and A. J. A. Meirelles, Fluid Phase Equilib. 366, 88 (2014). https://doi.org/10.1016/j.fluid.2014.01.004 |
| $C_{19}H_{40}O$ | 284.520 | 19 | 1-nonadecanol | 333.900 | 60.750 | 152.19 | 152.19 | L. Ventola, T. Calvet, M. A. Cuevas-Diarte, M. Ramirez, H. A. J. Oonk, D. Mondieig, and P. Negrier, Phys. Chem. Chem. Phys. 6, 1786 (2004). https://doi.org/10.1039/B313106H |
| $C_{20}H_{42}O$ | 298.547 | 20 | 1-eicosanol | 336.600 | 63.450 | 146.04 | 146.04 | G. Nichols, S. Kweskin, M. Frericks, S. Reiter, G. Wang, J. Orf, B. Carvallo, D. Hillesheim, and J. S. Chickos, J. Chem. Eng. Data 51, 475 (2006). https://doi.org/10.1021/je0503857 |
| $C_{22}H_{46}O$ | 326.600 | 22 | 1-docosanol | 345.200 | 72.050 | 142.59 | 142.59 | B. Tong, Z.-C. Tan, and S.-X. Wang, Wuli Huaxue Xuebao 24, 1699 (2008) |

| Molecular formula | Molecular weight (g mol$^{-1}$) | Carbon number | Compound name | $T_{fus}$ (K) | $T_{fus}$ (°C) | $\Delta_{fus}H$ (J g$^{-1}$) | Average $\Delta_{fus}H$ (J g$^{-1}$) | References |
|---|---|---|---|---|---|---|---|---|
| $C_{26}H_{54}O$ | 382.706 | 26 | 1-hexacosanol | 351.700 | 78.550 | 177.11 | 177.11 | G. Nichols, S. Kweskin, M. Frericks, S. Reiter, G. Wang, J. Orf, B. Carvallo, D. Hillesheim, and J. S. Chickos, J. Chem. Eng. Data 51, 475 (2006). https://doi.org/10.1021/je0503857 |

**Table S5.** Melting temperatures ($T_{fus}$) and total enthalpies of transition ($\Delta_{tot}H$) of fatty alcohols with carbon numbers between 12 and 26. $\Delta_{tot}H$ is the sum of the enthalpy of solid-solid transition and enthalpy of fusion. These data were used in Fig. 6 in the manuscript.

| Molecular formula | Molecular weight (g mol$^{-1}$) | Carbon number | $T_{fus}$ (K) | $T_{fus}$ (°C) | $\Delta_{tot}H$ (J g$^{-1}$) | References |
|---|---|---|---|---|---|---|
| $C_{12}H_{26}O$ | 186.334 | 12 | 297.53 | 24.38 | 206.40 | V. M. Egorov, V. A. Marikhin, and L. P. Myasnikova, Phys. Solid State 50, 126 (2008). https://doi.org/10.1134/S106378340801023X |
| $C_{13}H_{28}O$ | 200.361 | 13 | 304.7 | 31.55 | 223.50 | J. C. Van Miltenburg, G. J. K. van den Berg, and M. Ramirez, J. Chem. Eng. Data 48, 36 (2003). https://doi.org/10.1021/je025524o |
| $C_{14}H_{30}O$ | 214.387 | 14 | 311.2 | 38.05 | 219.27 | J. L. Zeng, Z. Cao, D. W. Yang, F. Xu, L. X. Sun, L. Zhang, and X. F. Zhang, J. Therm. Anal. Calorim. 95, 501 (2009). https://doi.org/10.1007/s10973-008-9274-x |
| $C_{15}H_{32}O$ | 228.414 | 15 | 316.6 | 43.45 | 239.61 | C. Mosselman and J. Mouric, J. Chem. Thermodyn. 6, 477–489 (1974). https://doi.org/10.1016/0021-9614(74)90009-3 |
| $C_{16}H_{34}O$ | 242.441 | 16 | 322.2 | 49.05 | 240.92 | Y. Kakiuchi, T. Sakurai, and T. Suzuki, J. Phys. Soc. Jpn. 5, 369 (1950). https://doi.org/10.1143/JPSJ.5.369 |
| $C_{17}H_{36}O$ | 256.467 | 17 | 325.3 | 52.15 | 247.20 | G. Nichols, S. Kweskin, M. Frericks, S. Reiter, G. Wang, J. Orf, B. Carvallo, D. Hillesheim, and J. S. Chickos, J. Chem. Eng. Data 51, 475 (2006). https://doi.org/10.1021/je0503857 |
| $C_{18}H_{38}O$ | 270.494 | 18 | 331.2 | 58.05 | 246.48 | J. C. van Miltenburg, H. A. J. Oonk, and L. Ventola, J. Chem. Eng. Data 46, 90 (2001). https://doi.org/10.1021/je000048s |
| $C_{19}H_{40}O$ | 284.520 | 19 | 334.5 | 61.35 | 254.53 | J. C. van Miltenburg, H. A. J. Oonk, and L. Ventola, J. Chem. Eng. Data 46, 90 (2001). https://doi.org/10.1021/je000048s |
| $C_{20}H_{42}O$ | 298.547 | 20 | 337 | 63.85 | 262.61 | J. C. van Miltenburg, H. A. J. Oonk, and L. Ventola, J. Chem. Eng. Data 46, 90 (2001). https://doi.org/10.1021/je000048s |
| $C_{22}H_{46}O$ | 326.600 | 22 | 344.5 | 71.35 | 253.52 | G. Nichols, S. Kweskin, M. Frericks, S. Reiter, G. Wang, J. Orf, B. Carvallo, D. Hillesheim, and J. S. Chickos, J. Chem. Eng. Data 51, 475 (2006). https://doi.org/10.1021/je0503857 |
| $C_{26}H_{54}O$ | 382.706 | 26 | 353.1 | 79.95 | 271.23 | A. A. Trapeznikov and T. A. Lomonosova, Zh. Fiz. Khim. 44, 2121–2128 (1970) |

**Table S6.** Melting temperatures ($T_{fus}$) and enthalpies of fusion ($\Delta_{fus}H$) of fatty acids with carbon numbers between 11 and 26. These data were used in Fig.1 and Fig. 7 in the manuscript.

| Molecular formula | Molecular weight (g mol$^{-1}$) | Carbon number | $T_{fus}$ (K) | $T_{fus}$ (°C) | $\Delta_{fus}H$ (J g$^{-1}$) | References |
|---|---|---|---|---|---|---|
| $C_{11}H_{22}O_2$ | 186.291 | 11 | 301.6 | 28.45 | 139.4591 | W. E. Garner and F. C. Randall, J. Chem. Soc., Trans. 125, 881 (1924). https://doi.org/10.1039/CT9242500881 |
| $C_{12}H_{24}O_2$ | 200.318 | 12 | 316.6 | 43.45 | 180.2137 | M. C. Costa, M. Sardo, M. P. Rolemberg, J. A. P. Coutinho, A. J. R. Meirelles, P. Ribeiro-Claro, and M. A. Krahenbuhl, Chem. Phys. Lipids 160, 85 (2009). https://doi.org/10.1016/j.chemphyslip.2009.05.004 |
| $C_{13}H_{26}O_2$ | 214.344 | 13 | 314.5 | 41.35 | 154.4244 | L. C. Liston, Y. Farnam, M. Krafcik, J. Weiss, K. Erk, and B. Y. Tao, Appl. Therm. Eng. 96, 501 (2016). https://doi.org/10.1016/j.applthermaleng.2015.11.007 |
| $C_{14}H_{28}O_2$ | 228.371 | 14 | 325.9 | 52.75 | 185.225 | J. Huang, S. Lu, X. Kong, S. Liu, and Y. Li, Materials 6, 4758 (2013). https://doi.org/10.3390/ma6104758 |
| $C_{15}H_{30}O_2$ | 242.398 | 15 | 325.7 | 52.55 | 171.2889 | J. C. van Miltenburg and H. A. J. Oonk, J. Chem. Eng. Data 50, 1348 (2005). https://doi.org/10.1021/je050065n |
| $C_{16}H_{32}O_2$ | 256.424 | 16 | 335.4 | 62.25 | 210.1987 | T. Inoue, Y. Hisatsugu, R. Ishikawa, and M. Suzuki, Chem. Phys. Lipids 127, 161 (2004). https://doi.org/10.1016/j.chemphyslip.2003.10.013 |
| $C_{17}H_{34}O_2$ | 270.451 | 17 | 334.3 | 61.15 | 189.7943 | J. S. Chickos and W. J. Hanshaw, Chem. Eng. Data 49, 77 (2004). https://doi.org/10.1021/je0301747 |
| $C_{18}H_{36}O_2$ | 284.477 | 18 | 342.8 | 69.65 | 222.1619 | F. O. Cedeno, M. M. Prieto, A. Espina, and J. R. Garcia, Thermochim. Acta 369, 39 (2001). https://doi.org/10.1016/S0040-6031(00)00752-8 |
| $C_{19}H_{38}O_2$ | 298.504 | 19 | 340.4 | 67.25 | 190.9523 | J. S. Chickos and W. J. Hanshaw, Chem. Eng. Data 49, 77 (2004). https://doi.org/10.1021/je0301747 |
| $C_{20}H_{40}O_2$ | 312.530 | 20 | 347.8 | 74.65 | 229.0977 | J. S. Chickos and W. J. Hanshaw, Chem. Eng. Data 49, 77 (2004). https://doi.org/10.1021/je0301747 |
| $C_{21}H_{42}O_2$ | 326.557 | 21 | 346.7 | 73.55 | 192.9219 | A. Sari, A. Bicer, and A. Karaipekli, Mater. Lett. 63, 1213 (2009). https://doi.org/10.1016/j.matlet.2009.02.045 |
| $C_{22}H_{44}O_2$ | 340.584 | 22 | 353.9 | 80.75 | 236.359 | V. M. Egorov, V. A. Marikhin, and L. P. Myasnikova, Polym. Sci., Ser. A 53, 906 (2011). https://doi.org/10.1134/S0965545X11100038 |
| $C_{23}H_{46}O_2$ | 354.610 | 23 | 352 | 78.85 | 211.4999 | J. S. Chickos and W. J. Hanshaw, Chem. Eng. Data 49, 77 (2004). https://doi.org/10.1021/je0301747 |
| $C_{24}H_{48}O_2$ | 368.637 | 24 | 356.5 | 83.35 | 229.223 | J. Wilson, C. Gobble, and J. Chickos, J. Chem. Eng. Data 60, 202 (2015). https://doi.org/10.1021/je5009729 |
| $C_{26}H_{52}O_2$ | 396.690 | 26 | 358.8 | 85.65 | 223.0962 | A. A. Aydin, Sol. Energy Mater. Sol. Cells 104, 102 (2012). https://doi.org/10.1016/j.solmat.2012.04.030 |

**Table S7.** Melting temperatures ($T_{fus}$) and enthalpies of fusion ($\Delta_{fus}H$) of esters with carbon number between 10 and 38. The data are sorted by ascending carbon numbers. For compounds with more than one data source, the average values of $T_{fus}$ and $\Delta_{fus}H$ were calculated. *A* = number of carbon atoms in the alkyl chain; *B* = number of carbon atoms in the ester root. These data were used in Fig.1, Fig. 3, and Fig. 8 in the manuscript.

| Molecular formula | Molecular weight (g mol$^{-1}$) | Carbon number | A | B | Compound name | $T_{fus}$ (K) | Average $T_{fus}$ (K) | $T_{fus}$ (°C) | Average $T_{fus}$ (°C) | $\Delta_{fus}H$ (J g$^{-1}$) | Average $\Delta_{fus}H$ (J g$^{-1}$) | References |
|---|---|---|---|---|---|---|---|---|---|---|---|---|
| $C_{10}H_{20}O_2$ | 172.270 | 10 | 5 | 5 | Pentyl valerate | 198.65 | | -74.5 | | 99.100 | | Stamatiou A, Obermeyer M, Fischer LJ, Schuetz P, Worlitschek J. Investigation of unbranched, saturated, carboxylic esters as phase change materials. Renew Energy 2017;108:401–9. https://doi.org/10.1016/j.renene.2017.02.056 |
| $C_{12}H_{24}O_2$ | 200.318 | 12 | 2 | 10 | Ethyl decanoate | 253.6 | 252.80 | -19.55 | -20.35 | 161.194 | 152.10 | T. M. T. Carvalho, L. M. P. F. Amaral, V. M. F. Morais, and M. D. M. C. Ribeiro da Silva, J. Chem. Thermodyn. 85, 129 (2015). https://doi.org/10.1016/j.jct.2015.01.012 |
| $C_{12}H_{24}O_2$ | 200.318 | 12 | | | | 252 | | -21.15 | | 143.000 | | Noël JA, Kahwaji S, White MA. Molecular Structure and Melting: Implications for Phase. Can J Chem 2017:cjc-2017-0578. https://doi.org/10.1139/cjc-2017-0578 |
| $C_{12}H_{24}O_2$ | 200.318 | 12 | 4 | 8 | Butyl octanoate | 230 | | -43.15 | | 140.000 | | Noël JA, Kahwaji S, White MA. Molecular Structure and Melting: Implications for Phase. Can J Chem 2017:cjc-2017-0578. https://doi.org/10.1139/cjc-2017-0578 |
| $C_{12}H_{24}O_2$ | 200.318 | 12 | 6 | 6 | Hexyl hexanoate | 217 | | -56.15 | | 108.000 | | Noël JA, Kahwaji S, White MA. Molecular Structure and Melting: Implications for Phase. Can J Chem 2017:cjc-2017-0578. https://doi.org/10.1139/cjc-2017-0578 |
| $C_{13}H_{26}O_2$ | 214.344 | 13 | 2 | 11 | Ethyl undecanoate | 259.2 | | -13.95 | | 168.701 | | V. M. Egorov, V. A. Marikhin, and L. P. Myasnikova, Polym. Sci., Ser. A 53, 906 (2011). https://doi.org/10.1134/S0965545X11100038 |
| $C_{14}H_{28}O_2$ | 228.371 | 14 | 2 | 12 | Ethyl dodecanoate | 271.5 | 272.35 | -1.65 | -0.80 | 176.905 | 167.42 | G. J. Maximo, N. D. D. Carereto, M. C. Costa, A. O. dos Santos, L. P. Cardoso, M. A. Krahenbuhl, and A. J. A. Meirelles, Fluid Phase Equilib. 366, 88 (2014). https://doi.org/10.1016/j.fluid.2014.01.004 |
| $C_{14}H_{28}O_2$ | 228.371 | 14 | | | | 271.5 | | -1.65 | | 40.767 | | M. D. Robustillo, D. F. Barbosa, A. J. de Almeida Meirelles, and P. de Alcantara Pessoa Filho, Fluid Phase Equilib. 361, 188 (2014). https://doi.org/10.1016/j.fluid.2013.10.024 |
| $C_{14}H_{28}O_2$ | 228.371 | 14 | | | | 273.2 | | 0.05 | | 157.945 | | E. S. Domalski and E. D. Hearing, J. Phys. Chem. Ref. Data 25, 1 (1996), and references therein. https://doi.org/10.1063/1.555985 |

| Molecular formula | Molecular weight (g mol$^{-1}$) | Carbon number | A | B | Compound name | $T_{fus}$ (K) | Average $T_{fus}$ (K) | $T_{fus}$ (°C) | Average $T_{fus}$ (°C) | $\Delta_{fus}H$ (J g$^{-1}$) | Average $\Delta_{fus}H$ (J g$^{-1}$) | References |
|---|---|---|---|---|---|---|---|---|---|---|---|---|
| $C_{15}H_{30}O_2$ | 242.398 | 15 | 2 | 13 | Ethyl tridecanoate | 272.4 | | -0.75 | | 167.906 | | W. E. Acree, Jr., Thermochim. Acta 219, 97 (1993); and references therein. https://doi.org/10.1016/0040-6031(93)80486-T |
| $C_{15}H_{30}O_2$ | 242.398 | 15 | 1 | 14 | Methyl tetradecanoate (methyl myristate) | 293.9 | 290.88 | 20.75 | 17.73 | 188.616 | 189.31 | E. S. Domalski and E. D. Hearing, J. Phys. Chem. Ref. Data 25, 1 (1996), and references therein. https://doi.org/10.1063/1.555985 |
| $C_{15}H_{30}O_2$ | 242.398 | 15 | 1 | 14 | | 287.85 | | 14.7 | | 190.000 | | Ravotti R, Fellmann O, Lardon N, Fischer LJ, Stamatiou A, Worlitschek J. Synthesis and Investigation of Thermal Properties of Highly Pure Carboxylic Fatty Esters to Be Used as PCM. Appl Sci 2018, Vol 8, Page 1069 2018;8:1069. https://doi.org/10.3390/APP8071069 |
| $C_{16}H_{32}O_2$ | 256.430 | 16 | 2 | 14 | Ethyl myristate | 287.25 | | 14.1 | | 173.400 | | Stamatiou A, Obermeyer M, Fischer LJ, Schuetz P, Worlitschek J. Investigation of unbranched, saturated, carboxylic esters as phase change materials. Renew Energy 2017;108:401–9. https://doi.org/10.1016/j.renene.2017.02.056 |
| $C_{16}H_{32}O_2$ | 256.430 | 16 | 4 | 12 | Butyl dodecanoate | 268.15 | | -5 | | 175.400 | | Stamatiou A, Obermeyer M, Fischer LJ, Schuetz P, Worlitschek J. Investigation of unbranched, saturated, carboxylic esters as phase change materials. Renew Energy 2017;108:401–9. https://doi.org/10.1016/j.renene.2017.02.056 |
| $C_{17}H_{34}O_2$ | 270.451 | 17 | 1 | 16 | Methyl hexadecanoate (methyl palmitate) | 300.61 | 303.55 | 27.46 | 30.40 | 188.352 | 214.68 | M. Benziane, K. Khimeche, A. Dahmani, S. Nezar, and D. Trache, J. Therm. Anal. Calorim. 112, 229 (2013). https://doi.org/10.1007/s10973-012-2654-2 |
| $C_{17}H_{34}O_2$ | 270.451 | 17 | | | | 302.2 | | 29.05 | | 231.466 | | E. S. Domalski and E. D. Hearing, J. Phys. Chem. Ref. Data 25, 1 (1996), and references therein. https://doi.org/10.1063/1.555985 |
| $C_{17}H_{34}O_2$ | 270.451 | 17 | | | | 302.2 | | 29.05 | | 214.827 | | A. M. King and W. E. Garner, J. Chem. Soc. 1936, 1372–1376. https://doi.org/10.1039/jr9360001372 |
| $C_{17}H_{34}O_2$ | 270.451 | 17 | | | | 302.2 | | 29.05 | | 198.927 | | M. W. Babich, S. W. Hwang, and R. D. Mounts, Thermochim. Acta 210, 83 (1992). https://doi.org/10.1016/0040-6031(92)80279-6 |
| $C_{17}H_{34}O_2$ | 270.451 | 17 | | | | 303.7 | | 30.55 | | 217.119 | | G. Gbabode, P. Negrier, D. Mondieig, E. M. Calvo, T. Calvet, and M. A. Cuevas-Diarte, |

| Molecular formula | Molecular weight (g mol$^{-1}$) | Carbon number | A | B | Compound name | $T_{fus}$ (K) | Average $T_{fus}$ (K) | $T_{fus}$ (°C) | Average $T_{fus}$ (°C) | $\Delta_{fus}H$ (J g$^{-1}$) | Average $\Delta_{fus}H$ (J g$^{-1}$) | References |
|---|---|---|---|---|---|---|---|---|---|---|---|---|
| | | | | | | | | | | | | Chem. - Eur. J. 13, 3150 (2007). https://doi.org/10.1002/chem.200600955 |
| $C_{17}H_{34}O_2$ | 270.451 | 17 | | | | 305.1 | | 31.95 | | 207.690 | | I. Abe, T. Kuroya, and K. Kusano, J. Jpn. Oil Chem. Soc. 34, 681 (1985). https://doi.org/10.5650/jos1956.34.681 |
| $C_{17}H_{34}O_2$ | 270.451 | 17 | | | | 305.2 | | 32.05 | | 207.062 | | W. E. Acree, Jr., Thermochim. Acta 219, 97 (1993); and references therein. https://doi.org/10.1016/0040-6031(93)80486-T |
| $C_{17}H_{34}O_2$ | 270.451 | 17 | | | | 307.2 | | 34.05 | | 252.024 | | G. Berchiesi, M. A. Bercuiesi, G. G. Lobbia, and D. Leonesi, Gazz. Chim. Ital. 106, 549–555 (1976) |
| $C_{17}H_{34}O_2$ | 270.451 | 17 | 7 | 10 | Heptyl decanoate | 269.25 | | -3.9 | | 147.600 | | Stamatiou A, Obermeyer M, Fischer LJ, Schuetz P, Worlitschek J. Investigation of unbranched, saturated, carboxylic esters as phase change materials. Renew Energy 2017;108:401–9. https://doi.org/10.1016/j.renene.2017.02.056 |
| $C_{18}H_{36}O_2$ | 284.477 | 18 | 1 | 17 | Methyl heptadecanoate | 304.2 | | 31.05 | | 169.082 | | M. D. Robustillo, D. F. Barbosa, A. J. de Almeida Meirelles, and P. de Alcantara Pessoa Filho, Fluid Phase Equilib. 361, 188 (2014). https://doi.org/10.1016/j.fluid.2013.10.024 |
| $C_{19}H_{38}O_2$ | 298.504 | 19 | 1 | 18 | Methyl stearate | 308.78 | | 35.63 | | 204.000 | | Ravotti R, Fellmann O, Lardon N, Fischer LJ, Stamatiou A, Worlitschek J. Synthesis and Investigation of Thermal Properties of Highly Pure Carboxylic Fatty Esters to Be Used as PCM. Appl Sci 2018, Vol 8, Page 1069 2018;8:1069. https://doi.org/10.3390/APP8071069 |
| $C_{19}H_{38}O_2$ | 298.504 | 19 | 5 | 14 | Pentyl tetradecanoate | 283.05 | | 9.9 | | 177.000 | | Ravotti R, Fellmann O, Lardon N, Fischer LJ, Stamatiou A, Worlitschek J. Analysis of bio-based fatty esters PCM's thermal properties and investigation of trends in relation to chemical structures. Appl Sci 2019;9:225. https://doi.org/10.3390/app9020225 |
| $C_{19}H_{38}O_2$ | 298.504 | 19 | 2 | 17 | Ethyl margarate (ethyl heptadecanoate) | 298.4 | | 25.25 | | 121.271 | | M. Benziane, K. Khimeche, A. Dahmani, S. Nezar, and D. Trache, J. Therm. Anal. Calorim. 112, 229 (2013). https://doi.org/10.1007/s10973-012-2654-2 |
| $C_{20}H_{40}O_2$ | 312.530 | 20 | 1 | 19 | Methyl nonadecanoate | 313.2 | 313.20 | 40.05 | 40.05 | 204.140 | 170.54 | M. M. Omar, J. Chem. Soc. C 2038–2040 (1967). https://doi.org/10.1039/j39670002038 |
| $C_{20}H_{40}O_2$ | 312.530 | 20 | | | | 313.2 | | 40.05 | | 136.947 | | E. Moreno, R. Cordobilla, T. Calvet, M. A. Cuevas-Diarte, G. Gbabode, P. Negrier, D. Mondieig, and H. A. J. Oonk, New J. Chem. 31, 947 (2007). https://doi.org/10.1039/b700551b |

| Molecular formula | Molecular weight (g mol$^{-1}$) | Carbon number | A | B | Compound name | $T_{fus}$ (K) | Average $T_{fus}$ (K) | $T_{fus}$ (°C) | Average $T_{fus}$ (°C) | $\Delta_{fus}H$ (J g$^{-1}$) | Average $\Delta_{fus}H$ (J g$^{-1}$) | References |
|---|---|---|---|---|---|---|---|---|---|---|---|---|
| $C_{21}H_{42}O_2$ | 326.557 | 21 | 2 | 19 | Ethyl nonadecanoate | 309 | | 35.85 | | 131.983 | | A. M. King and W. E. Garner, J. Chem. Soc. 1936, 1372–1376. https://doi.org/10.1039/jr9360001372 |
| $C_{21}H_{42}O_2$ | 326.557 | 21 | 1 | 20 | Methyl eicosanoate | 319.2 | 318.53 | 46.05 | 45.38 | 227.525 | 222.07 | G. Gbabode, P. Negrier, D. Mondieig, E. M. Calvo, T. Calvet, and M. A. Cuevas-Diarte, Chem. - Eur. J. 13, 3150 (2007). https://doi.org/10.1002/chem.200600955 |
| $C_{21}H_{42}O_2$ | 326.557 | 21 | | | | 319.2 | | 46.05 | | 225.688 | | M. M. Omar, J. Chem. Soc. C 2038–2040 (1967). https://doi.org/10.1039/j39670002038 |
| $C_{21}H_{42}O_2$ | 326.557 | 21 | | | | 317.2 | | 44.05 | | 213.000 | | Ravotti R, Fellmann O, Lardon N, Fischer LJ, Stamatiou A, Worlitschek J. Analysis of bio-based fatty esters PCM's thermal properties and investigation of trends in relation to chemical structures. Appl Sci 2019;9:225. https://doi.org/10.3390/app9020225 |
| $C_{21}H_{42}O_2$ | 326.557 | 21 | 5 | 16 | Ppentyl hexadecanoate | 292.75 | | 19.6 | | 187.000 | | Ravotti R, Fellmann O, Lardon N, Fischer LJ, Stamatiou A, Worlitschek J. Analysis of bio-based fatty esters PCM's thermal properties and investigation of trends in relation to chemical structures. Appl Sci 2019;9:225. https://doi.org/10.3390/app9020225 |
| $C_{22}H_{44}O_2$ | 340.584 | 22 | 6 | 16 | Hexylhexadecanoate | 287.6 | | 14.45 | | 164.982 | | M. M. Omar, J. Chem. Soc. C 2038–2040 (1967). https://doi.org/10.1039/j39670002038 |
| $C_{22}H_{44}O_2$ | 340.584 | 22 | 10 | 12 | Decyldodecanoate | 293.2 | | 20.05 | | 186.944 | | A. M. King and W. E. Garner, J. Chem. Soc. 1934, 1449. https://doi.org/10.1039/jr9340001449 |
| $C_{22}H_{44}O_2$ | 340.584 | 22 | 2 | 20 | Ethyleicosanoate | 313.6 | 314.30 | 40.45 | 41.15 | 201.419 | 201.45 | J. E. Baldvins and R. G. Weiss, Liq. Cryst. 26, 897 (1999). https://doi.org/10.1080/026782999204598 |
| $C_{22}H_{44}O_2$ | 340.584 | 22 | | | | 315 | | 41.85 | | 201.478 | | J. E. Baldvins and R. G. Weiss, Liq. Cryst. 26, 897 (1999). https://doi.org/10.1080/026782999204598 |
| $C_{22}H_{44}O_2$ | 340.584 | 22 | 1 | 21 | Methyl heneicosanoate | 321.2 | | 48.05 | | 220.504 | | J. A. Wilson and J. S. Chickos, J. Chem. Eng. Data 58, 322 (2013). https://doi.org/10.1021/je300902c |
| $C_{23}H_{46}O_2$ | 354.610 | 23 | 1 | 22 | Methyl docosanoate (methyl behenate) | 327.2 | 325.15 | 54.05 | 52.00 | 235.470 | 222.52 | G. Gbabode, P. Negrier, D. Mondieig, E. M. Calvo, T. Calvet, and M. A. Cuevas-Diarte, Chem. - Eur. J. 13, 3150 (2007). https://doi.org/10.1002/chem.200600955 |
| $C_{23}H_{46}O_2$ | 354.610 | 23 | | | | 327.2 | | 54.05 | | 232.086 | | A. M. King and W. E. Garner, J. Chem. Soc. 1936, 1372–1376. https://doi.org/10.1039/jr9360001372 |
| $C_{23}H_{46}O_2$ | 354.610 | 23 | | | | 321.05 | | 47.9 | | 200.000 | | Ravotti R, Fellmann O, Lardon N, Fischer LJ, Stamatiou A, Worlitschek J. Synthesis and Investigation of Thermal Properties of Highly Pure Carboxylic Fatty Esters to Be Used as PCM. Appl Sci 2018, Vol 8, Page 1069 |

| Molecular formula | Molecular weight (g mol$^{-1}$) | Carbon number | A | B | Compound name | $T_{fus}$ (K) | Average $T_{fus}$ (K) | $T_{fus}$ (°C) | Average $T_{fus}$ (°C) | $\Delta_{fus}H$ (J g$^{-1}$) | Average $\Delta_{fus}H$ (J g$^{-1}$) | References |
|---|---|---|---|---|---|---|---|---|---|---|---|---|
| | | | | | | | | | | | | 2018;8:1069. https://doi.org/10.3390/APP8071069 |
| $C_{23}H_{46}O_2$ | 354.610 | 23 | 5 | 18 | Pentyl octadecanoate | 298.32 | | 25.17 | | 151.000 | | Ravotti R, Fellmann O, Lardon N, Fischer LJ, Stamatiou A, Worlitschek J. Analysis of bio-based fatty esters PCM's thermal properties and investigation of trends in relation to chemical structures. Appl Sci 2019;9:225. https://doi.org/10.3390/app9020225 |
| $C_{24}H_{48}O_2$ | 368.637 | 24 | 2 | 22 | | 321 | 321.00 | 47.85 | 47.85 | 211.102 | 176.08 | E. S. Domalski and E. D. Hearing, J. Phys. Chem. Ref. Data 25, 1 (1996), and references therein. https://doi.org/10.1063/1.555985 |
| $C_{24}H_{48}O_2$ | 368.637 | 24 | | | | 321 | | 47.85 | | 141.060 | | M. M. Omar, J. Chem. Soc. C 2038–2040 (1967). https://doi.org/10.1039/j39670002038 |
| $C_{24}H_{48}O_2$ | 368.637 | 24 | 10 | 14 | | 298.35 | | 25.2 | | 196.000 | | Ravotti R, Fellmann O, Lardon N, Fischer LJ, Stamatiou A, Worlitschek J. Synthesis and Investigation of Thermal Properties of Highly Pure Carboxylic Fatty Esters to Be Used as PCM. Appl Sci 2018, Vol 8, Page 1069 2018;8:1069. https://doi.org/10.3390/APP8071069. |
| $C_{25}H_{50}O_2$ | 382.663 | 25 | 2 | 23 | Ethyl tricosanoate | 326 | | 52.85 | | 149.792 | | J. S. Chickos and W. J. Hanshaw, Chem. Eng. Data 49, 77 (2004). https://doi.org/10.1021/je0301747 |
| $C_{25}H_{50}O_2$ | 382.663 | 25 | 1 | 24 | Methyl tetracosanoate | 331.2 | | 58.05 | | 235.194 | | M. M. Omar, J. Chem. Soc. C 2038–2040 (1967). https://doi.org/10.1039/j39670002038 |
| $C_{25}H_{50}O_2$ | 382.663 | 25 | 5 | 20 | Pentyl eicosanoate | 308.6 | | 35.45 | | 189.000 | | Ravotti R, Fellmann O, Lardon N, Fischer LJ, Stamatiou A, Worlitschek J. Analysis of bio-based fatty esters PCM's thermal properties and investigation of trends in relation to chemical structures. Appl Sci 2019;9:225. https://doi.org/10.3390/app9020225 |
| $C_{26}H_{52}O_2$ | 396.690 | 26 | 16 | 10 | Hexadecyl decanoate | 302.6 | | 29.45 | | 186.292 | | E. S. Domalski and E. D. Hearing, J. Phys. Chem. Ref. Data 25, 1 (1996), and references therein. https://doi.org/10.1063/1.555985 |
| $C_{26}H_{52}O_2$ | 396.690 | 26 | 14 | 12 | Tetradecyl dodecanoate | 311.3 | | 38.15 | | 207.971 | | A. A. Aydin and H. Okutan, Sol. Energy Mater. Sol. Cells 95, 2752 (2011). https://doi.org/10.1016/j.solmat.2011.04.015 |
| $C_{26}H_{52}O_2$ | 396.690 | 26 | 1 | 15 | Methyl pentacosanoate | 332.2 | | 59.05 | | 231.919 | | J. Wilson, C. Gobble, and J. Chickos, J. Chem. Eng. Data 60, 202 (2015). https://doi.org/10.1021/je5009729 |
| $C_{26}H_{52}O_2$ | 396.690 | 26 | 10 | 16 | | 302.18 | | 29.03 | | 193.000 | | Ravotti R, Fellmann O, Lardon N, Fischer LJ, Stamatiou A, Worlitschek J. Synthesis and Investigation of Thermal Properties of Highly Pure Carboxylic Fatty Esters to Be Used as PCM. Appl Sci 2018, Vol 8, Page 1069 2018;8:1069. https://doi.org/10.3390/APP8071069 |

| Molecular formula | Molecular weight (g mol$^{-1}$) | Carbon number | A | B | Compound name | $T_{fus}$ (K) | Average $T_{fus}$ (K) | $T_{fus}$ (°C) | Average $T_{fus}$ (°C) | $\Delta_{fus}H$ (J g$^{-1}$) | Average $\Delta_{fus}H$ (J g$^{-1}$) | References |
|---|---|---|---|---|---|---|---|---|---|---|---|---|
| $C_{27}H_{54}O_2$ | 410.716 | 27 | 14 | 13 | Tetradecyl tridecanoate | 313.2 | | 40.05 | | 207.881 | | J. S. Chickos and W. J. Hanshaw, Chem. Eng. Data 49, 77 (2004). https://doi.org/10.1021/je0301747 |
| $C_{27}H_{54}O_2$ | 410.716 | 27 | 1 | 26 | Methyl hexacosanoate | 336.2 | | 63.05 | | 246.642 | | A. A. Aydin and H. Okutan, Sol. Energy Mater. Sol. Cells 95, 2752 (2011). https://doi.org/10.1016/j.solmat.2011.04.015 |
| $C_{27}H_{54}O_2$ | 410.716 | 27 | 5 | 22 | Pentyl docosanoate | 315.53 | | 42.38 | | 165.000 | | Ravotti R, Fellmann O, Lardon N, Fischer LJ, Stamatiou A, Worlitschek J. Analysis of bio-based fatty esters PCM's thermal properties and investigation of trends in relation to chemical structures. Appl Sci 2019;9:225. https://doi.org/10.3390/app9020225 |
| $C_{28}H_{56}O_2$ | 424.743 | 28 | 16 | 18 | Hexadecyl dodecanoate | 311.4 | | 38.25 | | 195.412 | | E. S. Domalski and E. D. Hearing, J. Phys. Chem. Ref. Data 25, 1 (1996), and references therein. https://doi.org/10.1063/1.555985 |
| $C_{28}H_{56}O_2$ | 424.743 | 28 | 14 | 14 | Tetradecyl tetradecanoate | 314.8 | 316.23 | 41.65 | 43.08 | 210.480 | 202.14 | A. A. Aydin and H. Okutan, Sol. Energy Mater. Sol. Cells 95, 2752 (2011). https://doi.org/10.1016/j.solmat.2011.04.015 |
| $C_{28}H_{56}O_2$ | 424.743 | 28 | | | | 317.65 | | 44.5 | | 193.800 | | Stamatiou A, Obermeyer M, Fischer LJ, Schuetz P, Worlitschek J. Investigation of unbranched, saturated, carboxylic esters as phase change materials. Renew Energy 2017;108:401–9. https://doi.org/10.1016/j.renene.2017.02.056 |
| $C_{28}H_{56}O_2$ | 424.743 | 28 | 1 | 27 | Methyl heptacosanoate | 336.2 | | 63.05 | | 237.085 | | A. A. Aydin, Sol. Energy Mater. Sol. Cells 104, 102 (2012). https://doi.org/10.1016/j.solmat.2012.04.030 |
| $C_{28}H_{56}O_2$ | 424.743 | 28 | 10 | 18 | | 309.37 | | 36.22 | | 192.000 | | Ravotti R, Fellmann O, Lardon N, Fischer LJ, Stamatiou A, Worlitschek J. Synthesis and Investigation of Thermal Properties of Highly Pure Carboxylic Fatty Esters to Be Used as PCM. Appl Sci 2018, Vol 8, Page 1069 2018;8:1069. https://doi.org/10.3390/APP8071069 |
| $C_{29}H_{58}O_2$ | 438.770 | 29 | 14 | 15 | Tetradecyl pentadecanoate | 318.6 | | 45.45 | | 214.805 | | J. S. Chickos and W. J. Hanshaw, Chem. Eng. Data 49, 77 (2004). https://doi.org/10.1021/je0301747 |
| $C_{29}H_{58}O_2$ | 438.770 | 29 | 1 | 18 | Methyl octacosanoate | 340.2 | | 67.05 | | 250.017 | | A. A. Aydin and H. Okutan, Sol. Energy Mater. Sol. Cells 95, 2752 (2011). https://doi.org/10.1016/j.solmat.2011.04.015 |
| $C_{30}H_{60}O_2$ | 452.796 | 30 | 18 | 12 | Octadecyl dodecanoate | 315.4 | | 42.25 | | 201.040 | | A. A. Aydin, Sol. Energy Mater. Sol. Cells 104, 102 (2012). https://doi.org/10.1016/j.solmat.2012.04.030 |
| $C_{30}H_{60}O_2$ | 452.796 | 30 | 14 | 16 | Tetradecyl hexadecanoate | 321.2 | | 48.05 | | 213.783 | | A. A. Aydin, Sol. Energy Mater. Sol. Cells 104, 102 (2012). https://doi.org/10.1016/j.solmat.2012.04.030 |

| Molecular formula | Molecular weight (g mol$^{-1}$) | Carbon number | A | B | Compound name | $T_{fus}$ (K) | Average $T_{fus}$ (K) | $T_{fus}$ (°C) | Average $T_{fus}$ (°C) | $\Delta_{fus}H$ (J g$^{-1}$) | Average $\Delta_{fus}H$ (J g$^{-1}$) | References |
|---|---|---|---|---|---|---|---|---|---|---|---|---|
| $C_{30}H_{60}O_2$ | 452.796 | 30 | 16 | 14 | Hexadecyl tetradecanoate | 322.6 | | 49.45 | | 225.267 | | A. A. Aydin, Sol. Energy Mater. Sol. Cells 113, 44 (2013). https://doi.org/10.1016/j.solmat.2013.01.024 |
| $C_{30}H_{60}O_2$ | 452.796 | 30 | 10 | 20 | Decyl eicosanoate | 314.04 | | 40.89 | | 232.000 | | Ravotti R, Fellmann O, Lardon N, Fischer LJ, Stamatiou A, Worlitschek J. Analysis of bio-based fatty esters PCM's thermal properties and investigation of trends in relation to chemical structures. Appl Sci 2019;9:225. https://doi.org/10.3390/app9020225 |
| $C_{30}H_{60}O_2$ | 452.796 | 30 | 12 | 18 | Lauryl stearate | 318.85 | | 45.7 | | 185.700 | | Stamatiou A, Obermeyer M, Fischer LJ, Schuetz P, Worlitschek J. Investigation of unbranched, saturated, carboxylic esters as phase change materials. Renew Energy 2017;108:401–9. https://doi.org/10.1016/j.renene.2017.02.056 |
| $C_{31}H_{62}O_2$ | 466.823 | 31 | 14 | 17 | Tetradecyl heptadecanoate | 319.9 | | 46.75 | | 217.192 | | A. A. Aydin and H. Okutan, Sol. Energy Mater. Sol. Cells 95, 2752 (2011). https://doi.org/10.1016/j.solmat.2011.04.015 |
| $C_{32}H_{64}O_2$ | 480.849 | 32 | 18 | 14 | Octadedcyl tetradecanoate | 322.1 | | 48.95 | | 203.536 | | A. A. Aydin and H. Okutan, Sol. Energy Mater. Sol. Cells 95, 2752 (2011). https://doi.org/10.1016/j.solmat.2011.04.015 |
| $C_{32}H_{64}O_2$ | 480.849 | 32 | 14 | 18 | Tetradecyl octadecanoate | 322.8 | | 49.65 | | 221.899 | | A. A. Aydin, Sol. Energy Mater. Sol. Cells 113, 44 (2013). https://doi.org/10.1016/j.solmat.2013.01.024 |
| $C_{32}H_{64}O_2$ | 480.849 | 32 | 16 | 16 | Hexadecyl hexadecanoate | 324.4 | 325.93 | 51.25 | 52.78 | 217.532 | 218.22 | A. A. Aydin, Sol. Energy Mater. Sol. Cells 104, 102 (2012). https://doi.org/10.1016/j.solmat.2012.04.030 |
| $C_{32}H_{64}O_2$ | 480.849 | 32 | | | | 327.45 | | 54.3 | | 218.900 | | Stamatiou A, Obermeyer M, Fischer LJ, Schuetz P, Worlitschek J. Investigation of unbranched, saturated, carboxylic esters as phase change materials. Renew Energy 2017;108:401–9. https://doi.org/10.1016/j.renene.2017.02.056 |
| $C_{32}H_{64}O_2$ | 480.849 | 32 | 2 | 30 | Ethyl triacontanoate | 341.5 | | 68.35 | | 155.974 | | E. S. Domalski and E. D. Hearing, J. Phys. Chem. Ref. Data 25, 1 (1996), and references therein. https://doi.org/10.1063/1.555985 |
| $C_{32}H_{64}O_2$ | 480.849 | 32 | 10 | 22 | | 317.95 | | 44.8 | | 193.000 | | Ravotti R, Fellmann O, Lardon N, Fischer LJ, Stamatiou A, Worlitschek J. Synthesis and Investigation of Thermal Properties of Highly Pure Carboxylic Fatty Esters to Be Used as PCM. Appl Sci 2018, Vol 8, Page 1069 2018;8:1069. https://doi.org/10.3390/APP8071069 |
| $C_{33}H_{66}O_2$ | 494.876 | 33 | 14 | 19 | Tetradecyl nonadecanoate | 323.4 | | 50.25 | | 203.223 | | A. A. Aydin and H. Okutan, Sol. Energy Mater. Sol. Cells 95, 2752 (2011). https://doi.org/10.1016/j.solmat.2011.04.015 |

| Molecular formula | Molecular weight (g mol$^{-1}$) | Carbon number | A | B | Compound name | $T_{fus}$ (K) | Average $T_{fus}$ (K) | $T_{fus}$ (°C) | Average $T_{fus}$ (°C) | $\Delta_{fus}H$ (J g$^{-1}$) | Average $\Delta_{fus}H$ (J g$^{-1}$) | References |
|---|---|---|---|---|---|---|---|---|---|---|---|---|
| $C_{34}H_{68}O_2$ | 508.903 | 34 | 16 | 18 | Hexadecyl octadecanoate | 327.8 | | 54.65 | | 214.186 | | A. A. Aydin, Sol. Energy Mater. Sol. Cells 104, 102 (2012). https://doi.org/10.1016/j.solmat.2012.04.030 |
| $C_{34}H_{68}O_2$ | 508.903 | 34 | 18 | 16 | Octadecyl hexadecanoate | 330.5 | | 57.35 | | 219.747 | | A. A. Aydin, Sol. Energy Mater. Sol. Cells 113, 44 (2013). https://doi.org/10.1016/j.solmat.2013.01.024 |
| $C_{34}H_{68}O_2$ | 508.903 | 34 | 12 | 22 | Lauryl behenate | 327.65 | | 54.5 | | 185.200 | | Stamatiou A, Obermeyer M, Fischer LJ, Schuetz P, Worlitschek J. Investigation of unbranched, saturated, carboxylic esters as phase change materials. Renew Energy 2017;108:401–9. https://doi.org/10.1016/j.renene.2017.02.056 |
| $C_{36}H_{72}O_2$ | 536.956 | 36 | 18 | 18 | Octadecyl octadecanoate | 332.4 | | 59.25 | | 214.934 | | A. A. Aydin, Sol. Energy Mater. Sol. Cells 104, 102 (2012). https://doi.org/10.1016/j.solmat.2012.04.030 |
| $C_{36}H_{72}O_2$ | 536.956 | 36 | 16 | 20 | Hexadecyl eicosanoate | 332.5 | | 59.35 | | 226.462 | | A. A. Aydin, Sol. Energy Mater. Sol. Cells 113, 44 (2013). https://doi.org/10.1016/j.solmat.2013.01.024 |
| $C_{38}H_{76}O_2$ | 565.009 | 38 | 18 | 20 | Octadecyl eicosanoate | 338.1 | | 64.95 | | 226.120 | | A. A. Aydin, Sol. Energy Mater. Sol. Cells 113, 44 (2013). https://doi.org/10.1016/j.solmat.2013.01.024 |

**Table S8.** Melting temperatures ($T_{fus}$) and enthalpies of fusion ($\Delta_{fus}H$) of diamides. These data were used in Fig.1 and Fig. 9 in the manuscript.

| Molecular formula | Molecular weight (g mol$^{-1}$) | Carbon number | Compound name | $T_{fus}$ (K) | $T_{fus}$ (°C) | $\Delta_{fus}H$ (J g$^{-1}$) | References |
|---|---|---|---|---|---|---|---|
| $C_8H_{16}N_2O_2$ | 172.228 | 8 | Suberamide | 493.23 | 220.08 | 338.9112 | E. Badea, G. Della Gata, D. D. Angelo, B. Brunetti, and Z. Reckova, J. Chem. Thermodyn. 38, 1546 (2006). https://doi.org/10.1016/j.jct.2006.04.004 |
| $C_9H_{18}N_2O_2$ | 186.254 | 9 | Azelamide | 450.41 | 177.26 | 295.4031 | E. Badea, G. Della Gata, D. D. Angelo, B. Brunetti, and Z. Reckova, J. Chem. Thermodyn. 38, 1546 (2006). https://doi.org/10.1016/j.jct.2006.04.004 |
| $C_{10}H_{20}N_2O_2$ | 200.28 | 10 | Sebacamide | 484.31 | 211.16 | 343.2694 | E. Badea, G. Della Gata, D. D. Angelo, B. Brunetti, and Z. Reckova, J. Chem. Thermodyn. 38, 1546 (2006). https://doi.org/10.1016/j.jct.2006.04.004 |
| $C_{11}H_{22}N_2O_2$ | 214.305 | 11 | Undecandiamide | 451.2 | 178.05 | 300.5069 | E. Badea, G. Della Gata, D. D. Angelo, B. Brunetti, and Z. Reckova, J. Chem. Thermodyn. 38, 1546 (2006). https://doi.org/10.1016/j.jct.2006.04.004 |
| $C_{12}H_{24}N_2O_2$ | 228.331 | 12 | Dodecandiamide | 466.1 | 192.95 | 322.7768 | E. Badea, G. Della Gata, D. D. Angelo, B. Brunetti, and Z. Reckova, J. Chem. Thermodyn. 38, 1546 (2006). https://doi.org/10.1016/j.jct.2006.04.004 |
| $C_{14}H_{28}N_2O_2$ | 256.384 | 14 | Tetradecandiamide | 469.3 | 196.15 | 302.0856 | E. Badea, G. Della Gata, D. D. Angelo, B. Brunetti, and Z. Reckova, J. Chem. Thermodyn. 38, 1546 (2006). https://doi.org/10.1016/j.jct.2006.04.004 |

**Table S9.** Melting temperatures ($T_{fus}$) and enthalpies of fusion ($\Delta_{fus}H$) of diamines. For compounds with more than one data source, the average values of $T_{fus}$ and $\Delta_{fus}H$ were calculated. These data were used in Fig.1 and Fig. 9 in the manuscript.

| Molecular formula | Molecular weight (g mol$^{-1}$) | Carbon number | Compound name | $T_{fus}$ (K) | Average $T_{fus}$ (K) | $T_{fus}$ (°C) | Average $T_{fus}$ (°C) | $\Delta_{fus}H$ (J g$^{-1}$) | Average $\Delta_{fus}H$ (J g$^{-1}$) | References |
|---|---|---|---|---|---|---|---|---|---|---|
| $C_8H_{20}N_2$ | 144.26 | 8 | Octane-1,8-diamine | 324.790 | 324.790 | 51.640 | 51.640 | 353.390 | 353.390 | L. Dall'acqua, G. Della Gatta, B. Nowicka, and P. Ferloni, J. Chem. Thermodyn. 34, 1 (2002). https://doi.org/10.1006/jcht.2001.0950 |
| $C_9H_{22}N_2$ | 158.286 | 9 | Nonane-1,9-diamine | 308.120 | 308.120 | 34.970 | 34.970 | 228.953 | 228.953 | L. Dall'acqua, G. Della Gatta, B. Nowicka, and P. Ferloni, J. Chem. Thermodyn. 34, 1 (2002). https://doi.org/10.1006/jcht.2001.0950 |
| $C_{10}H_{24}N_2$ | 172.312 | 10 | Decane-1,10-diamine | 332.880 | 332.880 | 59.730 | 59.730 | 335.496 | 335.496 | L. Dall'acqua, G. Della Gatta, B. Nowicka, and P. Ferloni, J. Chem. Thermodyn. 34, 1 (2002). https://doi.org/10.1006/jcht.2001.0950 |
| $C_{11}H_{26}N_2$ | 186.338 | 11 | Undecane-1,11-diamine | 313.640 | 313.640 | 40.490 | 40.490 | 258.026 | 258.026 | L. Dall'acqua, G. Della Gatta, B. Nowicka, and P. Ferloni, J. Chem. Thermodyn. 34, 1 (2002). https://doi.org/10.1006/jcht.2001.0950 |
| $C_{12}H_{28}N_2$ | 200.364 | 12 | 1,12-dodecanediamine | 340.500 | 341.150 | 67.350 | 68.000 | 334.890 | 334.890 | L. Dall'acqua, G. Della Gatta, B. Nowicka, and P. Ferloni, J. Chem. Thermodyn. 34, 1 (2002). https://doi.org/10.1006/jcht.2001.0950 |
| $C_{12}H_{28}N_2$ | 200.364 | 12 | 1,12-dodecanediamine | 341.800 | | 68.650 | | 334.890 | | K. Khimeche, Y. Boumrah, M. Benziane, and A. Dahmani, Thermochim. Acta 444, 166 (2006). https://doi.org/10.1016/j.tca.2006.03.011 |

**Table S10.** Melting temperatures ($T_{fus}$) and enthalpies of fusion ($\Delta_{fus}H$) of dinitriles. These data were used in Fig.1 and Fig. 9 in the manuscript.

| Molecular formula | Molecular weight (g mol$^{-1}$) | Carbon number | Compound name | $T_{fus}$ (K) | $T_{fus}$ (°C) | $\Delta_{fus}H$ (J g$^{-1}$) | References |
|---|---|---|---|---|---|---|---|
| $C_{10}H_{16}N_2$ | 164.248 | 10 | Sebaconitrile | 281.180 | 8.030 | 171.509 | Badea E, Blanco I, Della Gatta G. Fusion and solid-to-solid transitions of a homologous series of alkane-α,ω-dinitriles. J Chem Thermodyn 2007;39:1392–8. https://doi.org/10.1016/J.JCT.2007.03.005 |
| $C_{11}H_{18}N_2$ | 178.277 | 11 | Undecanonitrile | 266.140 | -7.010 | 145.897 | Badea E, Blanco I, Della Gatta G. Fusion and solid-to-solid transitions of a homologous series of alkane-α,ω-dinitriles. J Chem Thermodyn 2007;39:1392–8. https://doi.org/10.1016/J.JCT.2007.03.005 |
| $C_{12}H_{20}N_2$ | 192.304 | 12 | Dodecanedinitrile | 294.230 | 21.080 | 178.519 | Badea E, Blanco I, Della Gatta G. Fusion and solid-to-solid transitions of a homologous series of alkane-α,ω-dinitriles. J Chem Thermodyn 2007;39:1392–8. https://doi.org/10.1016/J.JCT.2007.03.005 |
| $C_{14}H_{24}N_2$ | 220.358 | 14 | Tetradecaneditrile | 309.550 | 36.400 | 182.294 | Badea E, Blanco I, Della Gatta G. Fusion and solid-to-solid transitions of a homologous series of alkane-α,ω-dinitriles. J Chem Thermodyn 2007;39:1392–8. https://doi.org/10.1016/J.JCT.2007.03.005 |

**Table S11.** Melting temperatures ($T_{fus}$) and enthalpies of fusion ($\Delta_{fus}H$) of diols. For compounds with carbon number ≥ 17, $\Delta_{fus}H$ data were not available from the cited references. These data were used in Fig.1 and Fig. 9 in the manuscript.

| Molecular formula | Molecular weight (g mol$^{-1}$) | Carbon number | Compound name | $T_{fus}$ (K) | $T_{fus}$ (°C) | $\Delta_{fus}H$ (J g$^{-1}$) | References |
|---|---|---|---|---|---|---|---|
| $C_{10}H_{22}O_2$ | 174.276 | 10 | 1,10-decanediol | 345.800 | 72.650 | 258.211 | Costa JCS, Santos LMNBF. Chain-Length Dependence of the Thermodynamic Behavior of Homologous α,ω-Disubstituted Alkanes. J Chem Eng Data 2019;64:2229–46. https://doi.org/10.1021/ACS.JCED.9B00125 |
| $C_{11}H_{24}O_2$ | 188.302 | 11 | 1,11-undecanediol | 334.100 | 60.950 | 243.757 | Costa JCS, Santos LMNBF. Chain-Length Dependence of the Thermodynamic Behavior of Homologous α,ω-Disubstituted Alkanes. J Chem Eng Data 2019;64:2229–46. https://doi.org/10.1021/ACS.JCED.9B00125 |
| $C_{12}H_{26}O_2$ | 202.328 | 12 | 1,12-dodecanediol | 352.900 | 79.750 | 267.882 | Costa JCS, Santos LMNBF. Chain-Length Dependence of the Thermodynamic Behavior of Homologous α,ω-Disubstituted Alkanes. J Chem Eng Data 2019;64:2229–46. https://doi.org/10.1021/ACS.JCED.9B00125 |
| $C_{13}H_{28}O_2$ | 216.354 | 13 | 1,13-tridecanediol | 350.300 | 77.150 | 253.289 | Costa JCS, Santos LMNBF. Chain-Length Dependence of the Thermodynamic Behavior of Homologous α,ω-Disubstituted Alkanes. J Chem Eng Data 2019;64:2229–46. https://doi.org/10.1021/ACS.JCED.9B00125 |
| $C_{14}H_{30}O_2$ | 230.38 | 14 | 1,14-tetradecanediol | 359.200 | 86.050 | 275.632 | Costa JCS, Santos LMNBF. Chain-Length Dependence of the Thermodynamic Behavior of Homologous α,ω-Disubstituted Alkanes. J Chem Eng Data 2019;64:2229–46. https://doi.org/10.1021/ACS.JCED.9B00125 |
| $C_{15}H_{32}O_2$ | 244.406 | 15 | 1,15-pentadecanediol | 361.200 | 88.050 | 267.178 | Costa JCS, Santos LMNBF. Chain-Length Dependence of the Thermodynamic Behavior of Homologous α,ω-Disubstituted Alkanes. J Chem Eng Data 2019;64:2229–46. https://doi.org/10.1021/ACS.JCED.9B00125 |
| $C_{16}H_{34}O_2$ | 258.432 | 16 | 1,16-hexadecanediol | 366.000 | 92.850 | 281.699 | Costa JCS, Santos LMNBF. Chain-Length Dependence of the Thermodynamic Behavior of Homologous α,ω-Disubstituted Alkanes. J Chem Eng Data 2019;64:2229–46. https://doi.org/10.1021/ACS.JCED.9B00125 |
| $C_{17}H_{36}O_2$ | 272.458 | 17 | 1,17-heptadecanediol | 367.300 | 94.150 | - | Costa JCS, Santos LMNBF. Chain-Length Dependence of the Thermodynamic Behavior of Homologous α,ω-Disubstituted Alkanes. J Chem Eng Data 2019;64:2229–46. https://doi.org/10.1021/ACS.JCED.9B00125 |
| $C_{18}H_{38}O_2$ | 286.484 | 18 | 1,18-octadecanediol | 371.500 | 98.350 | - | Costa JCS, Santos LMNBF. Chain-Length Dependence of the Thermodynamic Behavior of Homologous α,ω-Disubstituted Alkanes. J Chem Eng Data 2019;64:2229–46. https://doi.org/10.1021/ACS.JCED.9B00125 |
| $C_{19}H_{40}O_2$ | 300.51 | 19 | 1,19-nonadecanediol | 373.900 | 100.750 | - | Costa JCS, Santos LMNBF. Chain-Length Dependence of the Thermodynamic Behavior of Homologous α,ω-Disubstituted Alkanes. J Chem Eng Data 2019;64:2229–46. https://doi.org/10.1021/ACS.JCED.9B00125 |
| $C_{20}H_{42}O_2$ | 314.536 | 20 | 1,20-eicosanediol | 376.100 | 102.950 | - | Costa JCS, Santos LMNBF. Chain-Length Dependence of the Thermodynamic Behavior of Homologous α,ω-Disubstituted Alkanes. J Chem Eng Data 2019;64:2229–46. https://doi.org/10.1021/ACS.JCED.9B00125 |

**Table S12.** Melting temperatures ($T_{fus}$) and enthalpies of fusion ($\Delta_{fus}H$) of dioic acids. For compounds with carbon numbers 16 and 18, $\Delta_{fus}H$ data were not available from the cited references. These data were used in Fig.1 and Fig. 9 in the manuscript.

| Molecular formula | Molecular weight (g mol$^{-1}$) | Carbon number | Compound name | $T_{fus}$ (K) | $T_{fus}$ (°C) | $\Delta_{fus}H$ (J g$^{-1}$) | References |
|---|---|---|---|---|---|---|---|
| $C_{10}H_{18}O_4$ | 202.244 | 10 | Decanedioic acid | 404.000 | 130.850 | 201.737 | Costa JCS, Santos LMNBF. Chain-Length Dependence of the Thermodynamic Behavior of Homologous α,ω-Disubstituted Alkanes. J Chem Eng Data 2019;64:2229–46. https://doi.org/10.1021/ACS.JCED.9B00125 |
| $C_{11}H_{20}O_4$ | 216.27 | 11 | Undecanedioic acid | 385.000 | 111.850 | 183.567 | Costa JCS, Santos LMNBF. Chain-Length Dependence of the Thermodynamic Behavior of Homologous α,ω-Disubstituted Alkanes. J Chem Eng Data 2019;64:2229–46. https://doi.org/10.1021/ACS.JCED.9B00125 |
| $C_{12}H_{22}O_4$ | 230.296 | 12 | Dodecanedioic acid | 402.500 | 129.350 | 219.717 | Costa JCS, Santos LMNBF. Chain-Length Dependence of the Thermodynamic Behavior of Homologous α,ω-Disubstituted Alkanes. J Chem Eng Data 2019;64:2229–46. https://doi.org/10.1021/ACS.JCED.9B00125 |
| $C_{13}H_{24}O_4$ | 244.322 | 13 | Tridecanedioic acid | 386.300 | 113.150 | 202.192 | Costa JCS, Santos LMNBF. Chain-Length Dependence of the Thermodynamic Behavior of Homologous α,ω-Disubstituted Alkanes. J Chem Eng Data 2019;64:2229–46. https://doi.org/10.1021/ACS.JCED.9B00125 |
| $C_{14}H_{26}O_4$ | 258.348 | 14 | Tetradecanedioic acid | 397.300 | 124.150 | 218.697 | Costa JCS, Santos LMNBF. Chain-Length Dependence of the Thermodynamic Behavior of Homologous α,ω-Disubstituted Alkanes. J Chem Eng Data 2019;64:2229–46. https://doi.org/10.1021/ACS.JCED.9B00125 |
| $C_{16}H_{30}O_4$ | 286.41 | 16 | Hexadecanedioic acid | 395.400 | 122.250 | - | Costa JCS, Santos LMNBF. Chain-Length Dependence of the Thermodynamic Behavior of Homologous α,ω-Disubstituted Alkanes. J Chem Eng Data 2019;64:2229–46. https://doi.org/10.1021/ACS.JCED.9B00125 |
| $C_{18}H_{34}O_4$ | 314.5 | 18 | Octadecanedioic acid | 398.100 | 124.950 | - | Costa JCS, Santos LMNBF. Chain-Length Dependence of the Thermodynamic Behavior of Homologous α,ω-Disubstituted Alkanes. J Chem Eng Data 2019;64:2229–46. https://doi.org/10.1021/ACS.JCED.9B00125 |
